# Recent knowledge on hepatitis E virus in Suidae reservoirs and transmission routes to human

**DOI:** 10.1186/s13567-017-0483-9

**Published:** 2017-11-21

**Authors:** Nicole Pavio, Virginie Doceul, Eugénie Bagdassarian, Reimar Johne

**Affiliations:** 1 Animal Health Laboratory, UMR 1161 Virology, ANSES, Maisons-Alfort, France; 2 UMR 1161 Virology, INRA, Maisons-Alfort, France; 3 UMR 1161 Virology, PRES University Paris 12, National Veterinary School, Maisons-Alfort, France; 4 German Federal Institute for Risk Assessment, Berlin, Germany

## Abstract

Hepatitis E virus (HEV) causes self-limiting acute hepatitis in humans that can eventually result in acute liver failures or progress to chronic infections. While in tropical and sub-tropical areas, HEV infections are associated with important waterborne epidemics, in Northern countries, HEV infections are autochthonous with a zoonotic origin. In the past decade, it has become clear that certain HEV genotypes are zoonotic and that swine, and more generally Suidae, are the main reservoir. Zoonotic transmissions of the virus may occur via direct contact with infected pigs, wild boars or consumption of contaminated meat. This review describes the current knowledge on domestic and wild Suidae as reservoirs of HEV and the evidence of the different routes of HEV transmission between these animals and humans.

**Electronic supplementary material:**

The online version of this article (10.1186/s13567-017-0483-9) contains supplementary material, which is available to authorized users.

## Introduction

Hepatitis E virus (HEV) infection is highly prevalent in human worldwide, with more than 20 million infections each year (WHO). Clinical hepatitis E is usually self-limiting but some cases may evolve into fulminant hepatitis with poor prognosis. In some endemic regions, up to 30% of pregnant women infected by HEV during the 3rd trimester die from acute liver failure [1]. In Northern countries, chronic HEV infections are observed in immunosuppressive contexts, notably in solid organ transplant recipients [2]. In those patients, rapid progression toward cirrhosis is observed. Extrahepatic symptoms, such as neurological, kidney or hematological dysfunctions, have also been described. HEV is transmitted by the oral route and occasionally through the parenteral route after accidental transfusion of HEV positive blood donation. In endemic regions (tropical and subtropical areas), HEV is a waterborne disease associated with large epidemics related to accidental contamination of drinking water by sewage. In Northern countries, HEV is suspected to be mostly a foodborne disease transmitted through consumption of infected food products. HEV is unique among the hepatitis viruses since it is the only one possessing non-primate animal reservoirs and being a foodborne zoonosis. HEV is widespread in pig farms and consumption of pork products, specially containing pig liver, is associated with HEV infections [3]. HEV is also present in wild boars and consumption of game meat or hunting is associated with HEV exposure. Contact exposure with infected animals is a possible transmission route as well, since professional occupations with animal reservoirs (pig-farmer and -veterinarians, slaughterhouse- and forestry-workers, hunters) have a higher seroprevalence than the related general population. Interactions between wild and domestic suids occur and may contribute to the spread and maintenance of HEV in both reservoirs. This review aims at presenting the recent data on Suidae reservoirs, cross-contaminations between wild and domestic pigs and on vehicles of HEV exposure through contacts or consumption of food products from infected animals.

## Suidae reservoirs

**Figure 1:**
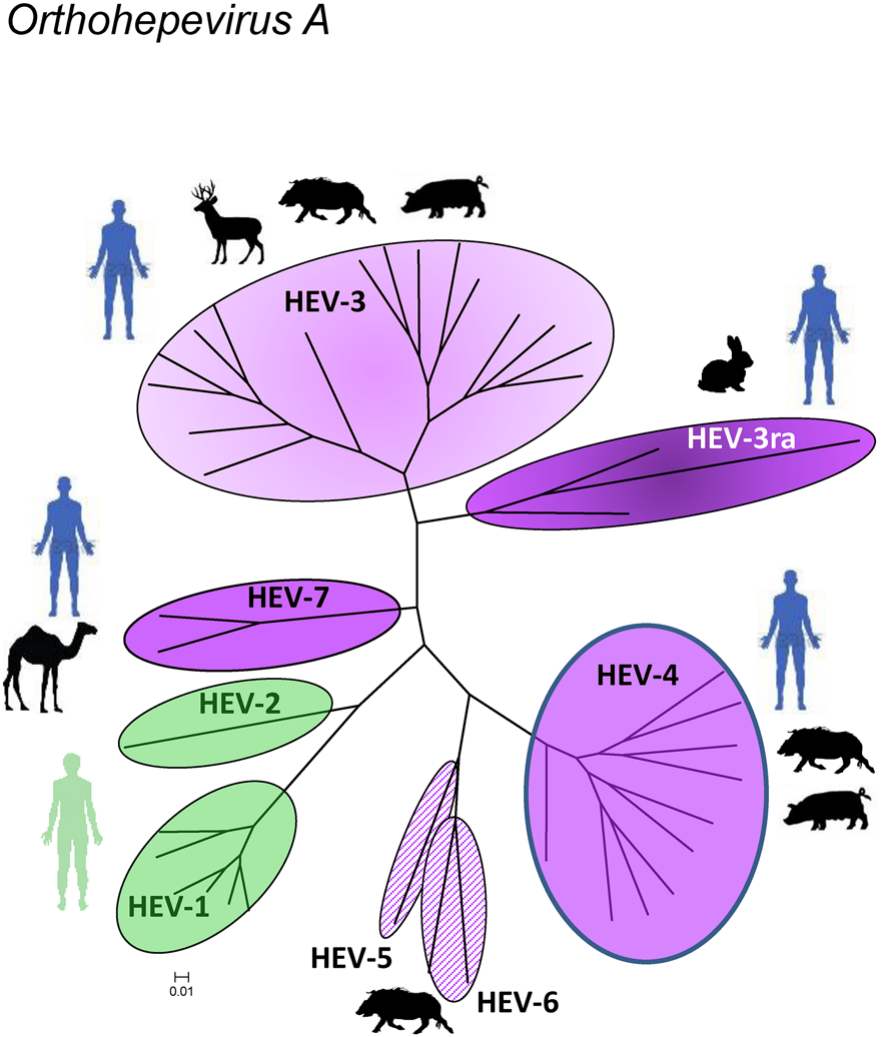
**Phylogenetic tree of HEV sequences within the species** ***Orthohepevirus A.*** Forty-one complete genomes or complete coding reference sequences available in the GenBank database and representative of each genotype sequences, as published by Smith et al. [44], were aligned using Muscle (MEGA6 [130]). The tree was obtained by applying the Neighbor-Joining method to a matrix of pairwise distances estimated using the Maximum Composite Likelihood (MCL) approach (MEGA6 [130]). The tree is drawn to scale, with branch lengths proportional to the number of substitutions per site. HEV genotypes are indicated for each group: HEV-1 to HEV-7 (HEV-3ra: rabbit subtype of HEV-3). Silhouettes of human or animal species indicate natural hosts. Human HEV are in green and zoonotic HEV in purple. HEV-5 and HEV-6 are in striped purple since their zoonotic potential remains to be proven.

HEV belongs to the Hepeviridae family which is divided into the genera *Orthohepevirus* and *Piscihepevirus* [3]. In the *Orthohepevirus* genus, four species (A to D) can be distinguished that are divided into several genotypes. Human and zoonotic HEV are classified into the Orthohepevirus species A, which includes seven genotypes. HEV-1 and HEV-2 infect humans only, whereas HEV-3 and HEV-4 can be found both in human and Suidae species (Figure 1). Within the genotype HEV-3, a separate branch corresponds to the HEV present in rabbit species (HEV-3ra), which also includes a closely related human strain (Figure 1). Two other genotypes, HEV-5 and HEV-6, have been described only in wild boars from Japan [4] (Figure 1). HEV-7 was first identified in dromedary camel [5] and then in one patient with chronic hepatitis after liver-transplantation [6].

HEV-3 is widely distributed and present in pigs from many geographic areas including Americas, Europe, Africa, Japan, south-east Asia and Oceania, whereas HEV-4 is described mainly in pigs from China, Japan and Indonesia [7].

### Domestic swine

#### HEV infection in domestic pigs

Since the discovery of swine HEV in 1997 [8], numerous publications have shown high prevalence of HEV in pig herds (up to 100%) all over the world. Each continent is concerned: Asia (China, India, Indonesia, Japan, Korea, Mongolia, Philippines, Taiwan, Thailand and Vietnam), Americas (Argentina, Bolivia, Brazil, Canada, Cuba and Mexico), Africa (Cameroon, Democratic Republic of Congo, Nigeria and Madagascar), Europe (Belgium, Czech Republic, Finland, France, Germany, Hungary, Italy, the Netherlands, Romania, Spain, Sweden, Switzerland and the United Kingdom) and Oceania (Australia, New Caledonia and New Zeeland) [7, 9–14]. Seroprevalences were estimated between 5 and 100% [7, 9–14]. Comparisons of HEV seroprevalences between countries are limited since sample collections (serum, occasionally meat juice) [15] and method of analysis are very diverse. Both commercial ELISA tests and in house assays were used to detect anti-HEV IgG and/or IgM. Viral prevalences are even more heterogeneous as they depend on the sampling strategies used: nature of the sample (blood, fecal, liver, other organs), age of the animals and method of analysis by conventional or real time RT-PCR that are still not standardized.

All studies converge toward an infection of pigs at an early age after the loss of maternal antibodies [16]. The peak of viral excretion in pig fecal samples is detected between 3 and 8 weeks after weaning [17], and then viral excretion decreases between 15 and 18 weeks of age [18] with the appearance of antibodies, IgM followed by IgG (seroconversion) [19]. The duration of the immunity acquired after HEV infection has not been estimated. Possible re-infection in case of transient decrease of immunity (sow after delivery or during co-infection) cannot be excluded. A loss of protection by a decrease of antibody or cellular response over time may also happen in older animals, especially in sows [20].

The presence of HEV RNA in serum samples (viremia) is less documented and seems to be less frequent than in liver or fecal samples [21]. Thus, the search for HEV RNA in blood or serum for diagnosis and prevalence studies may not be the best method to detect acute HEV infection in natural settings. Also, experimental infection of pigs shows that viremia can be influenced by the inoculation route [22]. Viral RNA was detected more frequently in the blood of infected animals after intravenous inoculation than after oral inoculation [22].

In both natural and experimental infections of pigs, rare histological signs of hepatitis are observed and infected animals do not show weight loss or hyperthermia. Thus, HEV in pig herds is not considered as an animal health threat. The main site of HEV multiplication is the liver but HEV can be found in other pork organs after experimental infections. HEV RNA or replicative forms of HEV RNA can be detected in extra-hepatic sites such as spleen, duodenum, jejunum, colon, lung, gastro-hepatic lymph nodes and muscles [22–24]. In one study, HEV was additionally detected in the lung [22]. Bouwknegt et al. reported the detection of HEV in tissues from three different muscles (Longissimus, Biceps femoris, Iliopsoas) in most of the inoculated and contact-infected pigs [23]. Generally, the highest detection rates and amounts of HEV RNA are found in the liver, whereas other organs show lower detection rates as well as lower HEV RNA concentrations. In these experimental models, several inoculation routes were used: intravenous, oral route and contact with inoculated animals. Again, depending on the inoculation route, HEV distribution was modulated, suggesting that experimental settings influence the outcome of the viral infection and may not completely reflect natural infection. Presence of HEV in muscle or other pig organs entering the food chain is of concern for food safety. Up to now, only few studies were conducted at slaughterhouse, on small sample size, to investigate the presence of HEV RNA in the organs of naturally infected pigs. In one of these studies, HEV RNA was searched in several organs such as loin, bladder or tonsils from selected animals (*n* = 43) [25]. HEV was detected in the bladder (10/43) and tonsils (3/43). No HEV-positive loin samples were observed [25]. In another study, detection of HEV RNA was investigated in the pork production chain at several steps: production (carcass dissection and liver removal), processing or point of sale. At the production phase (slaughterhouse), HEV RNA was found in liver, bile or fecal samples but also in pork lingual muscle with an estimated prevalence of HEV RNA of 2.7% (*n* = 112) [26]. At all steps, production, processing plant or point of sale, HEV RNA was amplified from workers’ hands and gloves and working surfaces, suggesting that cross contamination may occur [26]. Thus, further studies are needed to determine HEV distribution in pork organs during slaughtering.

Hepatitis E virus can persist in the farm environment [27] but survival parameters are mainly unknown. Experiments performed with HEV in cell culture supernatant indicate that infectious HEV can be detected after one month storage at room temperature and after more than 2 months storage at 4 °C [28]. The virus is mainly excreted fecally in pigs, leading to an accumulation of HEV in the environment of infected livestock. Thus, both contact between individuals and environmental exposure can play a role in HEV transmission. This was confirmed during the controlled inoculations of animals with HEV and transmission to contact pigs [29, 30]. Experimentally, a minimum load of 10^6^ copies of HEV RNA/g appears necessary to infect pigs per os and cause excretion of the virus and transmission to their congeners [29]. A mathematical model, taking into account three possible transmission pathways: direct or indirect contacts between the animals or the role played by the environment, was used to quantify HEV transmission within herds. The results obtained show that transmission by direct contact can be a factor of persistence of HEV in pig herds, however this way of transmission alone does not explain the high prevalences observed in herds. The major factor identified as playing a role in the spread and maintenance of infection in the population is the accumulation of the virus in the animal environment, leading to a continuous oro-fecal contamination process [29].

Environmental contaminations with HEV can also be observed in the vicinities of herds or slaughterhouse. HEV RNA was detected by nested RT-PCR in 32/452 samples from both inside and outside farm buildings, on trucks, and other objects sampled in the slaughterhouse yard, such as on a utility vehicle. According to the results of this study, the movements of trucks and utility vehicles might play an important role in HEV dissemination on a slaughterhouse site and throughout an entire network [31].

#### Risk factors associated with HEV infection within pig farms

A possible way to reduce human exposure through consumption of infected meat is to reduce the number of infected animals at slaughterhouse. To reach this goal, few studies have investigated factors associated with a high HEV prevalence at herd level. A retrospective survey was conducted in France in 90 farms previously selected for a prevalence survey of HEV performed at slaughterhouse [32]. A high risk of HEV infection of pork livers was associated with the early slaughtering of young animal, the genetic line of the female breeding stock, lack of biosecurity measures and the use of drinking water from a nearby source. High seroprevalences at the end of the rearing period were associated with excessive mixtures of post-weaning animals and low conditions of hygiene [33]. Critical points to consider include interactions between husbandry conditions, loss of passive immunity and hygiene.

In a study performed in six European countries (Czech Republic, Italy, the Netherlands, Spain, Portugal and the United Kingdom), HEV prevalence was determined in different types of herds: weaners, growers and fatteners. HEV RNA prevalence in pig fecal samples was high in all kinds of settings: weaners 8–30%, growers 20–44% and fatteners/finishers 8–73%. These data suggest that HEV positive fattener herds are at risk of delivering HEV positive pigs at slaughterhouse [34]. This risk can arise both from livers still infected with HEV or fecal cross-contaminations of carcasses. Analysis of livestock management was not performed to identify risk factors associated with positivity. Thus, further studies are needed to define measures to prevent such high HEV presence at the fattening stage.

Another study has focused on HEV seroprevalence in pigs from different farming systems in The Netherlands, including conventional, free-range, and organic farms. HEV-specific antibodies were detected in samples from all conventional, free-range and organic pig farms, indicating equal probability of introducing HEV for the different farming types. The estimated average within-herd seroprevalence was significantly higher for pigs from organic farms (89%) than for pigs from conventional farms (72%, *P* = 0.04) and close to significant for pigs from free-range farms (76%, *P* = 0.06) [35]. It seems that a higher probability of HEV infection is present in organic farm due to possible increase in animal contacts or longer exposure through environmental contamination [35]. It would be interesting to get information on the age of the pig sampled within each herd and the excretion profile of HEV in free-range and organic farms to evaluate the probability that these rearing systems deliver HEV positive pigs at slaughterhouse.

Another concern is the presence of other pig pathogens that may modulate the time-course of natural HEV infections within herds. Frequent co-circulation of HEV with immunomodulatory viruses such as Porcine Reproductive and Respiratory Syndrome virus (PRRSV) or Porcine circovirus-2 (PCV2), may influence HEV pathogenesis. A fatal disease associated with co-infection of HEV and PCV2 was recently described in piglets. General hyperthermia, hemorrhage, inflammatory cell infiltration and necrosis were observed in the tissues of dead animals [36]. In addition, experimental co-infection of pig with HEV and PRRSV demonstrated increased and prolonged excretion of HEV in co-infected pigs. This co-infection led to an increased length of HEV excretion estimated at 49 days compared to 9 days during infection with HEV alone. An exacerbated transmission, on average four times greater than in a single HEV infection, was also observed [37]. Multiple infections of pig by pathogens must be taken into account to prevent HEV propagation or long time excretion that may influence the presence of HEV at the time of slaughtering.

Hepatitis E virus infections have little impact on animal health, as the animals have no obvious symptoms; as a result, there is no surveillance or reporting of infected herds. The level of enzootic HEV infections is suspected to be extremely high. The persistence of the virus in farms is not yet well explained, but its resistance in the environment, the possibility of partial protection conferred by antibodies or the chronic infection of certain animals are factors that could make its eradication difficult. Drastic internal biosecurity measures as well as the control of undercurrent pathogens that may have an immunomodulatory action such as PRRSV would seem to be effective in limiting the spread of HEV to the porcine population. The effect of HEV vaccine prophylaxis in animals has been modelled and may reduce the number of HEV-positive animals at slaughter [38] and thus reduce human exposure via food consumption.

Even if control measures, such as good herd practice and vaccination against HEV, would lead to a reduced level of enzootic HEV in herds, maintaining HEV “low or free” herds would have to take into account the large presence of HEV in wildlife and in particular in wild boars.

### Wild fauna

Substantial evidences have been gathered in the past 20 years indicating that wild boar is an important reservoir of HEV. It is not surprising that wild boars (Sus scrofa) are also susceptible to HEV as they are closely related to domestic pigs (Sus scrofa domesticus). The first report suggesting that HEV can also infect wild boars comes from the detection of HEV antibodies in wild-caught pigs in Australia at the end of the 1990s [39]. A few years later, the first partial sequences of an HEV isolate originating from a wild boar were obtained in Japan [40]. Comparison of ORF2 partial sequences (298 nucleotides) showed that this strain was closely related to HEV-3 isolates previously found in Japanese patients and farm pigs [40]. The first full-genome HEV sequences recovered from a wild boar were published a few months later. Comparison with complete or near-complete HEV isolates showed that this strain belongs to HEV-3 and shares 99.7% nucleotide identity with other deer and human isolates [41]. To date, most of the HEV strains detected in wild boars belong to HEV-3. Several variants assigned to HEV-4 have also been found in wild boars in Japan [4, 42, 43]. In addition, HEV isolates (JBOAR135-Shiz09, wbJOY_06 and wbJNN_13) that have been only detected in wild boars have been characterized [42, 43]. These HEV strains have been assigned to two novel genotypes: HEV-5 (JBOAR135-Shiz09) and HEV-6 (wbJOY_06 and wbJNN_13). These 2 genotypes share less than 80% nucleotide identity with HEV-1 to -4 [42, 43]. The wbJNN_13 and wbJOY_06 isolates are 80.4% similar over their entire genome [42] and it has been suggested that they belong to two distinct subtypes within the HEV-6 genotype [44]. Up to now, no human case has been associated with these two genotypes, thus their zoonotic potential is not known.

Very few data are available on clinical signs caused by HEV in wild boar. In one study, no difference was found between the biometric characteristics (body length and weight) of HEV-infected and non-infected wild boars [45]. In another report, no clinical manifestations were observed in a viremic male wild boar negative for anti-HEV IgG [40]. Experimental infection of 3-month old wild boars with a wild boar HEV-3 strain via the intravenous route or by contact caused subtle clinical symptoms such as reduced feed intake and mild diarrhea that were concomitant with increased bile acids (BA), alanine aminotransferase (ALT) and gamma-glutamyl transferase (γGT) [46]. Mild lymphoplasmacytic hepatitis was also recorded. Higher viral loads of HEV were found in the feces of the infected wild boars in comparison to the feces of miniature pigs infected under the same conditions [46]. HEV RNA was also detected in the liver, gall bladder, small and large intestine and spleen of the infected wild boars. Chronic infections in two wild boars naturally infected with HEV-3 have also been reported [47]. In these two animals, viremia and/or viral shedding in the feces were detected for 12–16 weeks although high titres of anti-HEV antibodies were simultaneously present in their serum. No clinical signs or histopathological lesions evocative of hepatitis were recorded and HEV RNA was not detected in the liver or other tissues of the chronically-infected wild boars [47].

Several studies have been undertaken, mainly in Japan and Europe, to determine the prevalence of HEV antibodies and/or RNA in wild boars (Additional file 1). Seroprevalences ranging from 1.6 to 41.6% and from 4.9 to 57.4% were found in Japan and Europe, respectively. RNA prevalences of up to 10.3% in Japan and up to 68.2% in Europe were also reported. These data clearly show that HEV infection is widely present in wild boars in Japan and in Europe and that wild swine likely represent a reservoir of HEV in these areas. Several factors such as the geographical location [4, 14, 42, 43, 45, 48–54], the year of sampling [4, 42, 48, 55, 56], the wild boar density [53, 57, 58] and the managing conditions [48, 59] can impact HEV prevalence. A higher HEV seroprevalence is usually found in adults and subadults than in juvenile [14, 48, 49, 52, 55, 60, 61]. No difference was observed between male and female wild boars [43, 45, 48, 58, 59, 61–64]. The reported HEV RNA prevalences (Additional file 1) can also vary according to the wild boar sample specimen used as HEV RNA seems to be more frequently detected in bile followed by liver and serum [50], as found for domestic pigs [65].

Comparisons of data obtained using the same serological assay have highlighted lower HEV seroprevalences in wild boars than in domestic pigs sampled in the same region [14, 54, 55, 60, 64]. In addition, HEV RNA can be found in wild boars in all age groups including animals older than 2 years [40, 45, 48, 52, 58, 59, 61, 63, 66–69] whereas in domestic pigs, it is more often detected in young animals (2–6 months) [70–73]. These data suggest that HEV circulates at lower rates and more progressively in wild boars than in pigs. The fact that domestic pigs are reared under intensive conditions and that wild boars are free-ranging and gather at lower densities could explain these differences. It is also possible that in wild boars, a weak or short-lasting protective immunity against HEV leads to chronic infections or re-infections of older animals.

**Figure 2:**
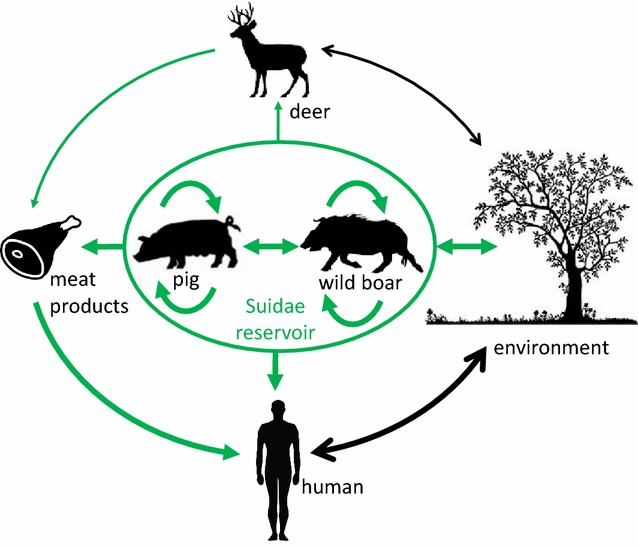
**Transmission and exposure routes of zoonotic HEV to humans.** Green arrow: proven transmission or exposure route; black arrow: suspected transmission or exposure route. The thickness of the arrows is proportional to the contribution of the species to the transmission or exposure route.

Hepatitis E virus has also been detected in deer. Anti-HEV antibodies and/or HEV RNA have been found in several deer species such as red deer, roe deer, sika deer, fallow deer and white-tailed deer in America (Mexico and Canada), Asia (China, Japan) and Europe (Czech Republic, Belgium, France, Germany, Hungary, Italy, The Netherlands, Spain, Sweden) [40, 56, 60, 66, 68, 74–83]. Anti-HEV antibodies were found in up to 62% (89/142) of white-tailed deer sampled in Mexico [74] but HEV seroprevalences ranging from 0 to 14% are more commonly found in several deer species. To date, all the HEV sequences found in deer belong to HEV-3 [41, 56, 60, 78, 79, 82–84] but very few sequences are available. All these studies suggest that HEV circulates in deer. However, lower HEV seroprevalences and HEV RNA detection rates are frequently found in deer compared to wild boars sampled within the same geographical region [40, 56, 60, 77, 81]. A recent study has also shown that lower viral loads are consistently found in livers from deer in comparison to wild boar [56]. This data suggest that deer are not a true reservoir of HEV but are infected accidentally by sharing the same habitat as wild boar. Wild swine therefore likely represent a source of spillover HEV infections for other wild animals and subsequently for humans (Figure 2).

### HEV transmission between domestic and wild Suidae

Experimental infections have shown that HEV can cross the domestic pig-wild boar barrier. Oral and intravenous inoculation of wild boar HEV strains to miniature or domestic pigs can lead to viremia, excretion of HEV in the feces and seroconversion [22, 46]. In addition, wild boars naturally infected or inoculated intravenously with HEV are able to transmit the virus by contact to domestic or miniature pigs [46, 47]. These data strongly suggest that transmission via the fecal–oral route between domestic and wild swine is possible and can occur naturally when pigs and wild boars are reared in close contact (Figure 2). Such interactions between domestic pigs and wild boars are common as some breeds of domestic pigs are raised outdoors in semi-open or open spaces in close contact with wildlife. These managing conditions exist worldwide, including in areas such as Corsica and Tuscany where a high HEV seroprevalence is found among wild boars [55, 61]. It is also important to consider the role of hybrid pigs in the epidemiology of HEV infection in swine. Wild boar can interbreed with domestic pigs, resulting in the birth of feral hybrid pigs that circulate freely but have a behavior that is more similar to domestic pigs (diurnal activity, more frequent and larger litter). These hybrid pigs are more likely to interact with domestic pigs and to gather at higher densities than wild boars. A study has shown that in Corsica, a high percentage of hybrid pigs (43.5%) has anti-HEV antibodies. This seroprevalence is lower than in domestic pigs (88%) but higher than in pure wild boars (26%) [55]. Hybrid pigs could then play an intermediate role in the transmission of HEV between domestic and wild swine.

Several studies have identified pig and wild boar strains of HEV-3 and HEV-4 sharing 90–98% homology based on entire/almost entire [58, 69] or partial [45, 55, 59, 63, 66, 67, 79, 85] sequences. For example, the comparison of full-length sequences has highlighted an identity of 96.9% between the sequences of a wild boar HEV-3 strain identified in Germany and a Mongolian pig strain [58]. A homology of 97.5% (partial ORF2 sequence) has been found between a wild boar and a domestic pig HEV-3 strain both isolated in Corsica [85]. Two wild boar HEV-3 strains isolated in Hungary were shown to be closer to pig strains than to each other (97–98% identity based on partial ORF2 sequences) [79]. In addition, a molecular evolutionary analysis has suggested that a subtype of HEV-3 has become endemic in Japan after the importation of infected pigs from Europe in the 1960s and was then transmitted from pigs to wild boars [86]. All these data suggest that dynamic exchanges of HEV-3 and HEV-4 between domestic pigs and wild boars have occurred. However, direct transmission of HEV between domestic pigs and wild boar in natural settings will only be proven by the identification of identical or near-identical (higher than 99%) strains in both animals. Moreover, the transmission potential of HEV-5 and HEV-6 between domestic and wild swine remains unknown as to date, these genotypes have only been detected in wild boars and attempt of experimental transmission of these two genotypes to pigs has not been reported yet.

As of now, it is still unclear whether cross-infection of HEV occurs between domestic and wild swine and contributes to the prevalence and dynamics of HEV infection in the swine population. Many studies need to be undertaken in order to better understand the implication of the wild boar reservoir in the epidemiology of HEV and its importance as a source of infection. It would be critical for example to determine the origin of HEV-5 and HEV-6 and whether these two genotypes are able to infect and cause diseases in domestic pigs, humans and other wild species such as deer. Other routes of HEV transmission between pigs and wild boars such as the contamination of surface water and the environment by wild boar droppings should also be considered and further explored (Figure 2). In addition, more studies are needed to determine whether chronic or recurrent HEV infections are common in wild boars as long-term shedding could facilitate the persistence of the virus in the wild boar population and environment, thus increasing the risk of transmission to nearby outdoor pig breeding and other wild species such as deer. It is still unclear whether HEV can be transmitted between swine and deer. Evidence of transmission between these species are rare and rely mainly on the identification of near-identical sequences in both species [41, 56].

## HEV in food products from Suidae and deer

Many reports describe the detection of HEV RNA in animal liver intended for human consumption and the data are summarized in Table 1. This includes reports from many European countries, but also from North, Central and South America as well as from Africa and Asia. As different methods with different sensitivities have been applied for analysis of the samples in these studies, a direct comparison of detection rates and genome concentrations is not possible. Nevertheless, the data may give some indications on the distribution of HEV in different types of food in the distinct geographical areas. The reported detection rates in pig liver ranged from 0 to 21%; however, the majority of studies report detection rates between 2 and 8%. Using quantitative analysis, HEV genome concentrations between 20 and 10^7^ RNA copies/g are described. For wild boar liver, studies from Europe and Japan are available, reporting detection rates between 2 and 38%. The HEV genome concentrations were between 40 and 10^8^ RNA copies/g. Investigations on liver samples from other animal species intended for human consumption are mainly available for several deer species. This includes successful HEV detection in red deer (detection rates 2–10%) and roe deer (detection rates 0–22%), whereas investigations of liver samples from fallow deer, Yezo deer and sika deer remained mostly negative so far. The HEV RNA amounts in liver of deer ranged between 12 and 2000 copies/g. Therefore, livers of pigs and wild boars have to be considered at high risk of containing the HEV genome and some of these livers can contain high viral genome amounts. Deer liver can also contain HEV RNA; however, the prevalence as well as the amount of viral genome in deer liver samples is markedly lower than for pigs and wild boars.

**Table 1:** **Summary of reports on detection of HEV RNA in liver, meat and meat products from animals intended for human consumption**

Animal species	Organ	Geographical area/Country	Detection rate	RNA log copies/g	References
Pig	Liver	Brazil	2/118 (2%)		[115]
		Burkina Faso	1/157 (1%)		[116]
		Cameroon	3/345 (1%)		[117]
		Canada	2/19 (10%)	1.3–1.6	[90]
		Canada	25/283 (9%)	3–6.7	[118]
		Canada	9/43 (21%)	3–7	[25]
		China	4/114 (4%)		[119]
		Czech Republic	2/40 (5%)		[26]
		France (Corsica)	2/24 (8%)		[55]
		France	128/3715 (4%)		[32]
		Germany	8/200 (4%)		[120]
		Hong Kong	7/479 (2%)		[121]
		India	2/240 (1%)		[122]
		Italy	2/33 (6%)		[26]
		Japan	12/243 (4.9%)		[123]
		Japan	0/110 (0%)		[124]
		Japan	4/390 (1%)		[125]
		Mexico	26/127 (20%)		[126]
		Spain	1/39 (3%)		[26]
		Thailand	3/1090 (1%)		[127]
		The Netherlands	4/62 (6%)		[98]
		United Kingdom	1/40 (3%)		[92]
		USA	14/127 (11%)		[97]
	Meat (muscle)	Canada	0/599 (0%)		[118]
		Canada	0/43 (0%)		[25]
		Czech Republic	1/40 (3%)		[26]
		Italy	2/33 (6%)		[26]
		Spain	0/39 (0%)		[26]
		Thailand	2/559 (1%)		[127]
		United Kingdom	0/40 (0%)		[92]
	Sausages (and other products) containing liver	Canada	36/76 (47%)	0.6–2.7	[90]
		France	68/394 (17.3%)	2.2–6.3	[87]
		France	22/70 (31%)	1.6–6.2	[91]
		Germany	11/50 (22%)		[89]
		Italy	11/68 (16%)	3.4–5.3	[88]
	Sausages without liver	Germany	14/70 (20%)		[89]
	Sausages (not specified)	Canada	0/35 (0%)		[90]
		Czech Republic	0/92 (0%)		[26]
		Italy	0/128 (0%)		[26]
		Spain	6/93 (6%)		[26]
		United Kingdom	6/63 (10%)		[92]
Wild boar	Liver	Belgium	4/61 (7%)		[60]
		Czech Republic	50/437 (11%)	7.3	[81]
		France	7/285 (3%)		[128]
		France	5/86 (6%)	1.6–8.1	[80]
		Germany	39/232 (17%)	7.4	[56]
		Germany	4/22 (18%)		[111]
		Germany	22/148 (15%)		[58]
		Germany	48/126 (38%)		[50]
		Hungary	8/75 (11%)		[78]
		Italy	7/372 (2%)		[62]
		Italy	55/164 (33%)		[63]
		Japan	19/552 (3%)		[4]
		Japan	7/39 (18%)		[129]
		Japan	1/33 (3%)		[40]
		The Netherlands	2/102 (2%)		[66]
	Meat (muscle)	Germany	29/232 (12%)	3.6	[56]
		Germany	1/22 (5%)		[111]
		The Netherlands	0/64 (0%)		[66]
	Sausages without liver	Germany	1/10 (10%)		[89]
Red deer	Liver	Belgium	1/29 (3%)		[60]
		France	2/62 (3%)	1.1-3.1	[80]
		Germany	2/83 (2%)	3.3	[56]
		Hungary	3/30 (10%)		[78]
		The Netherlands	1/39 (3%)		[66]
	Meat (muscle)	Germany	2/83 (2%)	2.7	[56]
		The Netherlands	2/39 (5%)		[66]
Roe deer	Liver	Belgium	0/27 (0%)		[60]
		Germany	5/78 (6%)	3.3	[56]
		Hungary	9/41 (22%)		[78]
		Italy	0/30 (0%)		[62]
		The Netherlands	0/8 (0%)		[66]
	Meat (muscle)	Germany	4/78 (5%)	2.7	[56]
		The Netherlands	0/6 (0%)		[66]
Fallow deer	Liver	Germany	0/22 (0%)		[56]
	Meat (muscle)	Germany	0/22 (0%)		[56]
Yezo deer	Liver	Japan	0/79 (0%)		[125]
Sika deer	Liver	Japan	0/132 (0%)		[40]

Some studies have investigated the presence of HEV RNA in muscle and meat samples. The detection rates ranged from 0 to 6% for pig muscle, from 0 to 12% for wild boar muscle, and from 0 to 5% for deer muscle. Only very few quantitative data are available, which report HEV genome amounts between 500 and 4000 copies/g in wild boar and deer muscles. In conclusion, a lower percentage of muscle samples of pigs, wild boars and deer, which nevertheless can reach up to 12%, can contain HEV RNA, in comparable low concentrations.

Meat products have also been analyzed for the presence of the HEV genome. Almost all of these studies have investigated products from pigs. Consistent high detection rates between 16 and 47% have been described for sausages or meat products containing pig liver [87, 88]. The observed detection rates, which are higher than those determined for pig livers, are mainly explained by the use of livers from a high number of animals for the production of sausages and the resulting mixing effect [89, 90]. The number of HEV genome in the liver-containing products ranged from 4 to 2 × 10^6^ copies/g [91]. HEV RNA was also found in sausages containing no liver (and in sausages where the presence or absence of liver was not known), but with more varying detection rates ranging from 0 to 20% [26, 92].

Detection of HEV RNA does not necessarily indicate the presence of infectious virus. Therefore, attempts have been made to demonstrate the infectivity of food items previously tested positive for HEV RNA. However, the measurement of HEV infectivity is difficult due to the lack of efficient cell culture models for HEV propagation [93]. Therefore, only a few studies are available investigating the presence of infectious HEV in organ or meat products from animals intended for human consumption. An A549 cell culture system was successfully used to demonstrate infectivity of HEV present in pig liver sold at retail in Japan [94]. Using a 3D cell culture model, HEV was isolated from porcine liver sausages from retail in France thus demonstrating the presence of infectious virus [95]. The presence of infectious HEV in commercially sold pig livers was also demonstrated by experimental inoculation of sample homogenates into pigs [96–98].

Stronger evidence for transmission of HEV by ingestion of food of animal origin comes from case reports and outbreak investigations. Colson et al. reported an outbreak of hepatitis E involving 7 persons in France, who ate liver sausages named figatelli [99]. Closely related HEV sequences were detected in the patients and local sausage samples. A similar case where identical HEV sequences were identified in a French hepatitis E patient and the leftover figatellu has been reported later [100]. In another outbreak that occurred on a French coastal island, 3 persons diseased with hepatitis E [101]. The epidemiological investigations pointed towards a spit-roasted piglet as the infection source. Identical HEV sequences were detected in the patients and in a liquid manure sample from the farm where the piglet was born. Investigation of an acute hepatitis E case in an immunodeficient patient identified pork meat as the source of infection by sequence comparison between HEV strains detected in the patient and in the meat [102]. Two case reports from Japan identified grilled meat from wild boar as the source of infection by demonstrating identical HEV sequences in the patient and the meat sample [103, 104]. Similarly, a wild boar liver was shown to contain HEV sequences that were identical to the ones found in two patients from Japan who ate the liver [105]. Four hepatitis E cases in Japan could also be linked to the consumption of meat from sika deer as identical HEV sequences were identified in the patients and the leftover meat [84].

## Contact exposure with Suidae

Transmission of HEV via contact with pigs or wild boars has been repeatedly suggested in Europe, Asia, Africa and Southern America. Most evidence for this transmission pathway comes from serological studies analyzing persons with occupational contact with animals compared to a control group (Additional file 2). For the investigation of HEV transmission from pigs, the exposed groups usually included pig farmers, slaughterers, butchers and swine veterinarians. In most of the studies, these groups show a higher HEV-specific antibody prevalence as compared to non-exposed persons. However, a direct comparison of the data from the different studies is not possible as different assays were used. In addition, factors other than the contact with pigs could differ between the exposed and non-exposed groups (e.g. their nutritional behavior) and may have influenced the results. Some of the studies found no significant differences in the seroprevalences between exposed and non-exposed people [106, 107]. However, as the majority of the studies demonstrate higher prevalences in persons who had been in contact with pigs, HEV transmission through this pathway seems to be common.

To investigate the transmission pathway of HEV from wild animals to humans, a number of serological studies with forest workers and hunters has been conducted (Additional file 2). Two studies investigating forest workers in Germany and France found higher HEV-specific antibody prevalences in these groups compared to the control groups [108, 109]. A study performed in Japan has identified a markedly higher seroprevalence in wild boar hunters (25.3%) as compared to a group of residents (5.5%) from the same geographical area [110]. In a similar study carried out in Germany, the HEV seroprevalence found in hunters was only slightly increased in comparison to the general German population [111]. However, a more detailed analysis of the data revealed that hunters who frequently used gloves during disemboweling of wild boars, had a significantly lower anti-HEV antibody prevalence as compared to hunters using gloves never or infrequently. The data of these studies indicate that transmission of HEV by contact with wild animals and especially with wild boars is likely.

In contrast to numerous studies describing the presence of antibody as indicator of virus transmission from pigs or wild boars to humans, evidence for induction of disease after transmission of HEV by contact is rare. Renou et al. have described a case of acute hepatitis E in a patient in France who kept a pet pig and had frequent contact with it and with its excretions [112]. By comparison of HEV strains detected in the patient and in the pig, nucleotide sequence identities between 92 and 98% were determined for different genome regions. Although the strains were not identical, both strains were more closely related to each other than to other HEV strains found in the same geographical region. The authors suspected that a distinct (minor) quasispecies of HEV from the pig was transmitted to the patient by direct contact or contact with the excretions. HEV transmission has also occurred during the surgical training of a surgeon on pigs, through contact with infected inner organs [113]. Direct contamination of a slaughterhouse worker has been reported as well, confirming that hepatitis E can be an occupationally acquired illness by means of the manipulation of infected organs from pigs [114].

## Conclusions

In the past decade, many studies have provided evidence that Suidae are the main reservoir of zoonotic HEV and that the virus is endemic in pig farms worldwide. A few studies have started to identify risk factors associated with HEV infection in pig herds. Nevertheless, many factors remain undetermined. For example, the influence of swine genetic backgrounds on the host susceptibility to HEV infection is still unknown. It is also unclear whether chronic infections can occur in swine and whether chronicity is associated with co-infections with other common pig pathogens or certain host factors linked to immunity. More efforts are then needed to determine more thoroughly the parameters involved in the accumulation and persistence of HEV in the breeding environment. Studies on the efficiency of disinfection regimes and vaccination to restrict HEV spread in the pig population would also be useful. Such investigations will help to understand the dynamics of HEV infection in pig herds and to identify control measures able to limit the appearance and persistence of the virus in breeding farms. A better characterization of the role of wildlife as a persistent source of contamination for the environment, other wild animals like deer, and pig herds is also required. In addition, the zoonotic potential of wild boar strains HEV-5 and HEV-6 need to be assessed experimentally to determine whether domestic swine and potentially humans are susceptible to these strains and might develop diseases. Prevention of zoonotic HEV infection will also benefit from a better monitoring of the presence of infectious HEV in pork-derived foods. In the past few years, cell culture models have been developed that allow an efficient replication of HEV. Such systems should be exploited to determine which meat products (produced or not with liver) are at risk of containing infectious viruses. Better recommendations could then be provided to consumers regarding handling and cooking of such foods.

## Additional files

**Additional file 1.** **Prevalence of HEV-specific antibodies and HEV RNA among wild boars.** Data from the literature (as referenced) are summarized in the table according to the geographic location, year of sampling, type of specimen used and the genotype detected.

**Additional file 2.** **Summary of reports on serological studies in people with occupational contact to animals.** Data from published studies (as referenced) investigating the prevalence of anti-HEV antibodies in different types of exposed and control groups in different countries are summarized in the table.

## References

[1] Pérez-Gracia MT, Suay-García B, Mateos-Lindemann ML (2017) Hepatitis E and pregnancy: current state. Rev Med Virol. 10.1002/rmv.1929. (in press) 10.1002/rmv.1929 28318080

[2] Kamar N, Garrouste C, Haagsma EB, Garrigue V, Pischke S, Chauvet C, Dumortier J, Cannesson A, Cassuto-Viguier E, Thervet E, Conti F, Lebray P, Dalton HR, Santella R, Kanaan N, Essig M, Mousson C, Radenne S, Roque-Afonso AM, Izopet J, Rostaing L (2011). Factors associated with chronic hepatitis in patients with hepatitis E virus infection who have received solid organ transplants. Gastroenterology.

[3] Doceul V, Bagdassarian E, Demange A, Pavio N (2016). Zoonotic hepatitis E virus: classification, animal reservoirs and transmission routes. Viruses.

[4] Sato Y, Sato H, Naka K, Furuya S, Tsukiji H, Kitagawa K, Sonoda Y, Usui T, Sakamoto H, Yoshino S, Shimizu Y, Takahashi M, Nagashima S, Jirintai Nishizawa T, Okamoto H (2011). A nationwide survey of hepatitis E virus (HEV) infection in wild boars in Japan: identification of boar HEV strains of genotypes 3 and 4 and unrecognized genotypes. Arch Virol.

[5] Woo PCY, Lau SKP, Teng JLL, Tsang AKL, Joseph M, Wong EYM, Tang Y, Sivakumar S, Xie J, Bai R, Wernery R, Wernery U, Yuen K-Y (2014). New hepatitis E virus genotype in camels, the Middle East. Emerg Infect Dis.

[6] Lee G-H, Tan B-H, Chi-Yuan Teo E, Lim SG, Dan Y-Y, Wee A, Aw PPK, Zhu Y, Hibberd ML, Tan C-K, Purdy MA, Teo C-G (2016). Chronic infection with camelid hepatitis E virus in a liver transplant recipient who regularly consumes camel meat and milk. Gastroenterology.

[7] Thiry D, Mauroy A, Pavio N, Purdy MA, Rose N, Thiry E, de Oliveira-Filho EF (2017). Hepatitis E virus and related viruses in animals. Transbound Emerg Dis.

[8] Meng XJ, Purcell RH, Halbur PG, Lehman JR, Webb DM, Tsareva TS, Haynes JS, Thacker BJ, Emerson SU (1997). A novel virus in swine is closely related to the human hepatitis E virus. Proc Natl Acad Sci USA.

[9] Liu X, Saito M, Sayama Y, Suzuki E, Malbas FF, Galang HO, Furuse Y, Saito M, Li T, Suzuki A, Oshitani H (2015). Seroprevalence and molecular characteristics of hepatitis E virus in household-raised pig population in the Philippines. BMC Vet Res.

[10] de la Caridad Montalvo Villalba MD, Owot JC, Benedito EC, Corrreia B, Corredor MB, Flaquet PP, Frometa SS, Wong MS, de la Rodríguez Lay LD (2013). Hepatitis E virus genotype 3 in humans and swine, Cuba. Infect Genet Evol.

[11] Merino-Ramos T, Martín-Acebes MA, Casal J, Saiz J-C, Loza-Rubio E (2016). Prevalence of hepatitis E virus (HEV) antibodies in Mexican pigs. Food Environ Virol.

[12] Owolodun OA, Gerber PF, Giménez-Lirola LG, Kwaga JKP, Opriessnig T (2014). First report of hepatitis E virus circulation in domestic pigs in Nigeria. Am J Trop Med Hyg.

[13] Aniţă A, Gorgan L, Aniţă D, Oşlobanu L, Pavio N, Savuţa G (2014). Evidence of hepatitis E infection in swine and humans in the East Region of Romania. Int J Infect Dis.

[14] Burri C, Vial F, Ryser-Degiorgis M-P, Schwermer H, Darling K, Reist M, Wu N, Beerli O, Schöning J, Cavassini M, Waldvogel A (2014). Seroprevalence of hepatitis E virus in domestic pigs and wild boars in Switzerland. Zoonoses Public Health.

[15] Wacheck S, Werres C, Mohn U, Dorn S, Soutschek E, Fredriksson-Ahomaa M, Märtlbauer E (2012). Detection of IgM and IgG against hepatitis E virus in serum and meat juice samples from pigs at slaughter in Bavaria, Germany. Foodborne Pathog Dis.

[16] Feng R, Zhao C, Li M, Harrison TJ, Qiao Z, Feng Y, Ma Z, Wang Y (2011). Infection dynamics of hepatitis E virus in naturally infected pigs in a Chinese farrow-to-finish farm. Infect Genet Evol.

[17] Kantala T, Heinonen M, Oristo S, von Bonsdorff C-H, Maunula L (2015). Hepatitis E virus in young pigs in Finland and characterization of the isolated partial genomic sequences of genotype 3 HEV. Foodborne Pathog Dis.

[18] McCreary C, Martelli F, Grierson S, Ostanello F, Nevel A, Banks M (2008). Excretion of hepatitis E virus by pigs of different ages and its presence in slurry stores in the United Kingdom. Vet Rec.

[19] Pavio N, Meng X-J, Renou C (2010). Zoonotic hepatitis E: animal reservoirs and emerging risks. Vet Res.

[20] Casas M, Cortés R, Pina S, Peralta B, Allepuz A, Cortey M, Casal J, Martín M (2011). Longitudinal study of hepatitis E virus infection in Spanish farrow-to-finish swine herds. Vet Microbiol.

[21] Grierson S, Heaney J, Cheney T, Morgan D, Wyllie S, Powell L, Smith D, Ijaz S, Steinbach F, Choudhury B, Tedder RS (2015). Prevalence of hepatitis E virus infection in pigs at the time of slaughter, United Kingdom, 2013. Emerg Infect Dis.

[22] Thiry D, Rose N, Mauroy A, Paboeuf F, Dams L, Roels S, Pavio N, Thiry E (2017). Susceptibility of pigs to zoonotic hepatitis E virus genotype 3 isolated from a wild boar. Transbound Emerg Dis.

[23] Bouwknegt M, Rutjes SA, Reusken CBEM, Stockhofe-Zurwieden N, Frankena K, de Jong MCM, de Roda Husman AM, van der Poel WHM (2009). The course of hepatitis E virus infection in pigs after contact-infection and intravenous inoculation. BMC Vet Res.

[24] Williams TP, Kasorndorkbua C, Halbur PG, Haqshenas G, Guenette DK, Toth TE, Meng XJ (2001). Evidence of extrahepatic sites of replication of the hepatitis E virus in a swine model. J Clin Microbiol.

[25] Leblanc D, Poitras E, Gagné M-J, Ward P, Houde A (2010). Hepatitis E virus load in swine organs and tissues at slaughterhouse determined by real-time RT-PCR. Int J Food Microbiol.

[26] Di Bartolo I, Diez-Valcarce M, Vasickova P, Kralik P, Hernandez M, Angeloni G, Ostanello F, Bouwknegt M, Rodríguez-Lázaro D, Pavlik I, Ruggeri FM (2012). Hepatitis E virus in pork production chain in Czech Republic, Italy, and Spain, 2010. Emerg Infect Dis.

[27] Kasorndorkbua C, Opriessnig T, Huang FF, Guenette DK, Thomas PJ, Meng X-J, Halbur PG (2005). Infectious swine hepatitis E virus is present in pig manure storage facilities on United States farms, but evidence of water contamination is lacking. Appl Environ Microbiol.

[28] Johne R, Trojnar E, Filter M, Hofmann J (2016). Thermal stability of hepatitis E virus estimated by a cell culture method. Appl Environ Microbiol.

[29] Andraud M, Dumarest M, Cariolet R, Aylaj B, Barnaud E, Eono F, Pavio N, Rose N (2013). Direct contact and environmental contaminations are responsible for HEV transmission in pigs. Vet Res.

[30] Bouwknegt M, Teunis PFM, Frankena K, de Jong MCM, de Roda Husman AM (2011). Estimation of the likelihood of fecal-oral HEV transmission among pigs. Risk Anal.

[31] Nantel-Fortier N, Letellier A, Lachapelle V, Fravalo P, L’Homme Y, Brassard J (2016). Detection and phylogenetic analysis of the hepatitis E virus in a Canadian swine production network. Food Environ Virol.

[32] Rose N, Lunazzi A, Dorenlor V, Merbah T, Eono F, Eloit M, Madec F, Pavio N (2011). High prevalence of hepatitis E virus in French domestic pigs. Comp Immunol Microbiol Infect Dis.

[33] Walachowski S, Dorenlor V, Lefevre J, Lunazzi A, Eono F, Merbah T, Eveno E, Pavio N, Rose N (2014). Risk factors associated with the presence of hepatitis E virus in livers and seroprevalence in slaughter-age pigs: a retrospective study of 90 swine farms in France. Epidemiol Infect.

[34] Berto A, Backer JA, Mesquita JR, Nascimento MSJ, Banks M, Martelli F, Ostanello F, Angeloni G, Di Bartolo I, Ruggeri FM, Vasickova P, Diez-Valcarce M, Hernandez M, Rodriguez-Lazaro D, van der Poel WHM (2012). Prevalence and transmission of hepatitis E virus in domestic swine populations in different European countries. BMC Res Notes.

[35] Rutjes SA, Bouwknegt M, van der Giessen JW, de Roda Husman AM, Reusken CBEM (2014). Seroprevalence of hepatitis E virus in pigs from different farming systems in The Netherlands. J Food Prot.

[36] Yang Y, Shi R, She R, Mao J, Zhao Y, Du F, Liu C, Liu J, Cheng M, Zhu R, Li W, Wang X, Soomro MH (2015). Fatal disease associated with swine hepatitis E virus and porcine circovirus 2 co-infection in four weaned pigs in China. BMC Vet Res.

[37] Salines M, Barnaud E, Andraud M, Eono F, Renson P, Bourry O, Pavio N, Rose N (2015). Hepatitis E virus chronic infection of swine co-infected with porcine reproductive and respiratory syndrome virus. Vet Res.

[38] Backer JA, Berto A, McCreary C, Martelli F, van der Poel WHM (2012). Transmission dynamics of hepatitis E virus in pigs: estimation from field data and effect of vaccination. Epidemics.

[39] Chandler JD, Riddell MA, Li F, Love RJ, Anderson DA (1999). Serological evidence for swine hepatitis E virus infection in Australian pig herds. Vet Microbiol.

[40] Sonoda H, Abe M, Sugimoto T, Sato Y, Bando M, Fukui E, Mizuo H, Takahashi M, Nishizawa T, Okamoto H (2004). Prevalence of hepatitis E virus (HEV) infection in wild boars and deer and genetic identification of a genotype 3 HEV from a boar in Japan. J Clin Microbiol.

[41] Takahashi K, Kitajima N, Abe N, Mishiro S (2004). Complete or near-complete nucleotide sequences of hepatitis E virus genome recovered from a wild boar, a deer, and four patients who ate the deer. Virology.

[42] Takahashi M, Nishizawa T, Nagashima S, Jirintai S, Kawakami M, Sonoda Y, Suzuki T, Yamamoto S, Shigemoto K, Ashida K, Sato Y, Okamoto H (2014). Molecular characterization of a novel hepatitis E virus (HEV) strain obtained from a wild boar in Japan that is highly divergent from the previously recognized HEV strains. Virus Res.

[43] Hara Y, Terada Y, Yonemitsu K, Shimoda H, Noguchi K, Suzuki K, Maeda K (2014). High prevalence of hepatitis E virus in wild boar (Sus scrofa) in Yamaguchi Prefecture, Japan. J Wildl Dis.

[44] Smith DB, Simmonds P, Izopet J, Oliveira-Filho EF, Ulrich RG, Johne R, Koenig M, Jameel S, Harrison TJ, Meng X-J, Okamoto H, Van der Poel WHM, Purdy MA (2016). Proposed reference sequences for hepatitis E virus subtypes. J Gen Virol.

[45] Martelli F, Caprioli A, Zengarini M, Marata A, Fiegna C, Di Bartolo I, Ruggeri FM, Delogu M, Ostanello F (2008). Detection of hepatitis E virus (HEV) in a demographic managed wild boar (Sus scrofa scrofa) population in Italy. Vet Microbiol.

[46] Schlosser J, Eiden M, Vina-Rodriguez A, Fast C, Dremsek P, Lange E, Ulrich RG, Groschup MH (2014). Natural and experimental hepatitis E virus genotype 3-infection in European wild boar is transmissible to domestic pigs. Vet Res.

[47] Schlosser J, Vina-Rodriguez A, Fast C, Groschup MH, Eiden M (2015). Chronically infected wild boar can transmit genotype 3 hepatitis E virus to domestic pigs. Vet Microbiol.

[48] Michitaka K, Takahashi K, Furukawa S, Inoue G, Hiasa Y, Horiike N, Onji M, Abe N, Mishiro S (2007). Prevalence of hepatitis E virus among wild boar in the Ehime area of western Japan. Hepatol Res Off J Jpn Soc Hepatol.

[49] Carpentier A, Chaussade H, Rigaud E, Rodriguez J, Berthault C, Boué F, Tognon M, Touzé A, Garcia-Bonnet N, Choutet P, Coursaget P (2012). High hepatitis E virus seroprevalence in forestry workers and in wild boars in France. J Clin Microbiol.

[50] Adlhoch C, Wolf A, Meisel H, Kaiser M, Ellerbrok H, Pauli G (2009). High HEV presence in four different wild boar populations in East and West Germany. Vet Microbiol.

[51] Martinelli N, Pavoni E, Filogari D, Ferrari N, Chiari M, Canelli E, Lombardi G (2015). Hepatitis E virus in wild boar in the central northern part of Italy. Transbound Emerg Dis.

[52] Caruso C, Modesto P, Bertolini S, Peletto S, Acutis PL, Dondo A, Robetto S, Mignone W, Orusa R, Ru G, Masoero L (2015). Serological and virological survey of hepatitis E virus in wild boar populations in northwestern Italy: detection of HEV subtypes 3e and 3f. Arch Virol.

[53] Larska M, Krzysiak MK, Jabłoński A, Kęsik J, Bednarski M, Rola J (2015). Hepatitis E virus antibody prevalence in wildlife in Poland. Zoonoses Public Health.

[54] Ivanova A, Tefanova V, Reshetnjak I, Kuznetsova T, Geller J, Lundkvist Å, Janson M, Neare K, Velström K, Jokelainen P, Lassen B, Hütt P, Saar T, Viltrop A, Golovljova I (2015). Hepatitis E virus in domestic pigs, wild boars, pig farm workers, and hunters in Estonia. Food Environ Virol.

[55] Jori F, Laval M, Maestrini O, Casabianca F, Charrier F, Pavio N (2016). Assessment of domestic pigs, wild boars and feral hybrid pigs as reservoirs of hepatitis E virus in Corsica, France. Viruses.

[56] Anheyer-Behmenburg HE, Szabo K, Schotte U, Binder A, Klein G, Johne R (2017). Hepatitis E virus in wild boars and spillover infection in red and roe deer, Germany, 2013–2015. Emerg Infect Dis.

[57] Boadella M, Ruiz-Fons JF, Vicente J, Martín M, Segalés J, Gortazar C (2012). Seroprevalence evolution of selected pathogens in Iberian wild boar. Transbound Emerg Dis.

[58] Schielke A, Sachs K, Lierz M, Appel B, Jansen A, Johne R (2009). Detection of hepatitis E virus in wild boars of rural and urban regions in Germany and whole genome characterization of an endemic strain. Virol J.

[59] de Deus N, Peralta B, Pina S, Allepuz A, Mateu E, Vidal D, Ruiz-Fons F, Martín M, Gortázar C, Segalés J (2008). Epidemiological study of hepatitis E virus infection in European wild boars (Sus scrofa) in Spain. Vet Microbiol.

[60] Thiry D, Mauroy A, Saegerman C, Licoppe A, Fett T, Thomas I, Brochier B, Thiry E, Linden A (2017). Belgian wildlife as potential zoonotic reservoir of hepatitis E virus. Transbound Emerg Dis.

[61] Mazzei M, Nardini R, Verin R, Forzan M, Poli A, Tolari F (2015). Serologic and molecular survey for hepatitis E virus in wild boar (Sus scrofa) in Central Italy. New Microbes New Infect.

[62] Serracca L, Battistini R, Rossini I, Mignone W, Peletto S, Boin C, Pistone G, Ercolini R, Ercolini C (2015). Molecular investigation on the presence of hepatitis e virus (HEV) in wild game in North-Western Italy. Food Environ Virol.

[63] Montagnaro S, De Martinis C, Sasso S, Ciarcia R, Damiano S, Auletta L, Iovane V, Zottola T, Pagnini U (2015). Viral and antibody prevalence of hepatitis E in European wild boars (Sus scrofa) and hunters at zoonotic risk in the Latium region. J Comp Pathol.

[64] Sakano C, Morita Y, Shiono M, Yokota Y, Mokudai T, Sato-Motoi Y, Noda A, Nobusawa T, Sakaniwa H, Nagai A, Kabeya H, Maruyama S, Yamamoto S, Sato H, Kimura H (2009). Prevalence of hepatitis E virus (HEV) infection in wild boars (Sus scrofa leucomystax) and pigs in Gunma Prefecture, Japan. J Vet Med Sci.

[65] de Deus N, Seminati C, Pina S, Mateu E, Martín M, Segalés J (2007). Detection of hepatitis E virus in liver, mesenteric lymph node, serum, bile and faeces of naturally infected pigs affected by different pathological conditions. Vet Microbiol.

[66] Rutjes SA, Lodder-Verschoor F, Lodder WJ, van der Giessen J, Reesink H, Bouwknegt M, de Roda Husman AM (2010). Seroprevalence and molecular detection of hepatitis E virus in wild boar and red deer in The Netherlands. J Virol Methods.

[67] Mesquita JR, Oliveira RMS, Coelho C, Vieira-Pinto M, Nascimento MSJ (2014). Hepatitis E virus in sylvatic and captive wild boar from Portugal. Transbound Emerg Dis.

[68] Roth A, Lin J, Magnius L, Karlsson M, Belák S, Widén F, Norder H (2016). Markers for ongoing or previous hepatitis E virus infection are as common in wild ungulates as in humans in Sweden. Viruses.

[69] Nishizawa T, Takahashi M, Endo K, Fujiwara S, Sakuma N, Kawazuma F, Sakamoto H, Sato Y, Bando M, Okamoto H (2005). Analysis of the full-length genome of hepatitis E virus isolates obtained from wild boars in Japan. J Gen Virol.

[70] Wu J-C, Chen C-M, Chiang T-Y, Tsai W-H, Jeng W-J, Sheen I-J, Lin C-C, Meng X-J (2002). Spread of hepatitis E virus among different-aged pigs: two-year survey in Taiwan. J Med Virol.

[71] Seminati C, Mateu E, Peralta B, de Deus N, Martin M (2008). Distribution of hepatitis E virus infection and its prevalence in pigs on commercial farms in Spain. Vet J.

[72] Leblanc D, Ward P, Gagné M-J, Poitras E, Müller P, Trottier Y-L, Simard C, Houde A (2007). Presence of hepatitis E virus in a naturally infected swine herd from nursery to slaughter. Int J Food Microbiol.

[73] Takahashi M, Nishizawa T, Miyajima H, Gotanda Y, Iita T, Tsuda F, Okamoto H (2003). Swine hepatitis E virus strains in Japan form four phylogenetic clusters comparable with those of Japanese isolates of human hepatitis E virus. J Gen Virol.

[74] Medrano C, Boadella M, Barrios H, Cantú A, García Z, de la Fuente J, Gortazar C (2012). Zoonotic pathogens among white-tailed deer, northern Mexico, 2004–2009. Emerg Infect Dis.

[75] Weger S, Elkin B, Lindsay R, Bollinger T, Crichton V, Andonov A (2016). Hepatitis E virus seroprevalence in free-ranging deer in Canada. Transbound Emerg Dis.

[76] Zhang X-X, Qin S-Y, Zhang Y, Meng Q-F, Jiang J, Yang G-L, Zhao Q, Zhu X-Q (2015). First report of hepatitis E virus infection in sika deer in China. Biomed Res Int.

[77] Kukielka D, Rodriguez-Prieto V, Vicente J, Sánchez-Vizcaíno JM (2016). Constant Hepatitis E Virus (HEV) Circulation in wild boar and red deer in Spain: an increasing concern source of HEV zoonotic transmission. Transbound Emerg Dis.

[78] Forgách P, Nowotny N, Erdélyi K, Boncz A, Zentai J, Szucs G, Reuter G, Bakonyi T (2010). Detection of hepatitis E virus in samples of animal origin collected in Hungary. Vet Microbiol.

[79] Reuter G, Fodor D, Forgách P, Kátai A, Szucs G (2009). Characterization and zoonotic potential of endemic hepatitis E virus (HEV) strains in humans and animals in Hungary. J Clin Virol.

[80] Lhomme S, Top S, Bertagnoli S, Dubois M, Guerin J-L, Izopet J (2015). Wildlife reservoir for hepatitis E virus, Southwestern France. Emerg Infect Dis.

[81] Kubankova M, Kralik P, Lamka J, Zakovcik V, Dolanský M, Vasickova P (2015) Prevalence of hepatitis E virus in populations of wild animals in comparison with animals bred in game enclosures. Food Environ Virol 7:159–163 10.1007/s12560-015-9189-1 25771162

[82] Boadella M, Casas M, Martín M, Vicente J, Segalés J, de la Fuente J, Gortázar C (2010). Increasing contact with hepatitis E virus in red deer, Spain. Emerg Infect Dis.

[83] Di Bartolo I, Ponterio E, Angeloni G, Morandi F, Ostanello F, Nicoloso S, Ruggeri FM (2017). Presence of hepatitis E virus in a RED deer (*Cervus elaphus*) population in Central Italy. Transbound Emerg Dis.

[84] Tei S, Kitajima N, Takahashi K, Mishiro S (2003). Zoonotic transmission of hepatitis E virus from deer to human beings. Lancet.

[85] Pavio N, Laval M, Maestrini O, Casabianca F, Charrier F, Jori F (2016). Possible Foodborne transmission of hepatitis E virus from domestic pigs and wild boars from Corsica. Emerg Infect Dis.

[86] Nakano T, Takahashi K, Arai M, Okano H, Kato H, Ayada M, Okamoto H, Mishiro S (2013). Identification of European-type hepatitis E virus subtype 3e isolates in Japanese wild boars: molecular tracing of HEV from swine to wild boars. Infect Genet Evol.

[87] Pavio N, Merbah T, Thébault A (2014). Frequent hepatitis E virus contamination in food containing raw pork liver, France. Emerg Infect Dis.

[88] Di Bartolo I, Angeloni G, Ponterio E, Ostanello F, Ruggeri FM (2015). Detection of hepatitis E virus in pork liver sausages. Int J Food Microbiol.

[89] Szabo K, Trojnar E, Anheyer-Behmenburg H, Binder A, Schotte U, Ellerbroek L, Klein G, Johne R (2015). Detection of hepatitis E virus RNA in raw sausages and liver sausages from retail in Germany using an optimized method. Int J Food Microbiol.

[90] Mykytczuk O, Harlow J, Bidawid S, Corneau N, Nasheri N (2017). Prevalence and molecular characterization of the hepatitis E virus in retail pork products marketed in Canada. Food Environ Virol.

[91] Martin-Latil S, Hennechart-Collette C, Delannoy S, Guillier L, Fach P, Perelle S (2016). Quantification of hepatitis E virus in naturally-contaminated pig liver products. Front Microbiol.

[92] Berto A, Martelli F, Grierson S, Banks M (2012). Hepatitis E virus in pork food chain, United Kingdom, 2009-2010. Emerg Infect Dis.

[93] Cook N, D’Agostino M, Clarke E, Johne R (2016) FSA Project FS301014: A critical review of approaches to assess the infectivity of hepatitis E virus 10.1007/s12560-017-9303-7 28470455

[94] Takahashi H, Tanaka T, Jirintai S, Nagashima S, Takahashi M, Nishizawa T, Mizuo H, Yazaki Y, Okamoto H (2012). A549 and PLC/PRF/5 cells can support the efficient propagation of swine and wild boar hepatitis E virus (HEV) strains: demonstration of HEV infectivity of porcine liver sold as food. Arch Virol.

[95] Berto A, Van der Poel WHM, Hakze-van der Honing R, Martelli F, La Ragione RM, Inglese N, Collins J, Grierson S, Johne R, Reetz J, Dastjerdi A, Banks M (2013). Replication of hepatitis E virus in three-dimensional cell culture. J Virol Methods.

[96] Feagins AR, Opriessnig T, Guenette DK, Halbur PG, Meng XJ (2008). Inactivation of infectious hepatitis E virus present in commercial pig livers sold in local grocery stores in the United States. Int J Food Microbiol.

[97] Feagins AR, Opriessnig T, Guenette DK, Halbur PG, Meng X-J (2007). Detection and characterization of infectious Hepatitis E virus from commercial pig livers sold in local grocery stores in the USA. J Gen Virol.

[98] Bouwknegt M, Lodder-Verschoor F, van der Poel WHM, Rutjes SA, de Roda Husman AM (2007). Hepatitis E virus RNA in commercial porcine livers in The Netherlands. J Food Prot.

[99] Colson P, Borentain P, Queyriaux B, Kaba M, Moal V, Gallian P, Heyries L, Raoult D, Gerolami R (2010). Pig liver sausage as a source of hepatitis E virus transmission to humans. J Infect Dis.

[100] Renou C, Roque-Afonso A-M, Afonso A-MR, Pavio N (2014). Foodborne transmission of hepatitis E virus from raw pork liver sausage, France. Emerg Infect Dis.

[101] Guillois Y, Abravanel F, Miura T, Pavio N, Vaillant V, Lhomme S, Le Guyader FS, Rose N, Le Saux J-C, King LA, Izopet J, Couturier E (2016). High proportion of asymptomatic infections in an outbreak of hepatitis E associated with a spit-roasted piglet, France, 2013. Clin Infect Dis.

[102] Riveiro-Barciela M, Mínguez B, Gironés R, Rodriguez-Frías F, Quer J, Buti M (2015). Phylogenetic demonstration of hepatitis E infection transmitted by pork meat ingestion. J Clin Gastroenterol.

[103] Masuda J-I, Yano K, Tamada Y, Takii Y, Ito M, Omagari K, Kohno S (2005). Acute hepatitis E of a man who consumed wild boar meat prior to the onset of illness in Nagasaki, Japan. Hepatol Res.

[104] Li T-C, Chijiwa K, Sera N, Ishibashi T, Etoh Y, Shinohara Y, Kurata Y, Ishida M, Sakamoto S, Takeda N, Miyamura T (2005). Hepatitis E virus transmission from wild boar meat. Emerg Infect Dis.

[105] Matsuda H, Okada K, Takahashi K, Mishiro S (2003). Severe hepatitis E virus infection after ingestion of uncooked liver from a wild boar. J Infect Dis.

[106] De Sabato L, Di Bartolo I, Montomoli E, Trombetta C, Ruggeri FM, Ostanello F (2017). Retrospective study evaluating seroprevalence of hepatitis E virus in blood donors and in swine veterinarians in Italy (2004). Zoonoses Public Health.

[107] Hinjoy S, Nelson KE, Gibbons RV, Jarman RG, Mongkolsirichaikul D, Smithsuwan P, Fernandez S, Labrique AB, Patchanee P (2013). A cross-sectional study of hepatitis E virus infection in healthy people directly exposed and unexposed to pigs in a rural community in northern Thailand. Zoonoses Public Health.

[108] Dremsek P, Wenzel JJ, Johne R, Ziller M, Hofmann J, Groschup MH, Werdermann S, Mohn U, Dorn S, Motz M, Mertens M, Jilg W, Ulrich RG (2012). Seroprevalence study in forestry workers from eastern Germany using novel genotype 3- and rat hepatitis E virus-specific immunoglobulin G ELISAs. Med Microbiol Immunol.

[109] Chaussade H, Rigaud E, Allix A, Carpentier A, Touzé A, Delzescaux D, Choutet P, Garcia-Bonnet N, Coursaget P (2013). Hepatitis E virus seroprevalence and risk factors for individuals in working contact with animals. J Clin Virol.

[110] Toyoda K, Furusyo N, Takeoka H, Murata M, Sawayama Y, Hayashi J (2008). Epidemiological study of hepatitis E virus infection in the general population of Okinawa, Kyushu, Japan. J Gastroenterol Hepatol.

[111] Schielke A, Ibrahim V, Czogiel I, Faber M, Schrader C, Dremsek P, Ulrich RG, Johne R (2015). Hepatitis E virus antibody prevalence in hunters from a district in Central Germany, 2013: a cross-sectional study providing evidence for the benefit of protective gloves during disembowelling of wild boars. BMC Infect Dis.

[112] Renou C, Cadranel J-F, Bourlière M, Halfon P, Ouzan D, Rifflet H, Carenco P, Harafa A, Bertrand JJ, Boutrouille A, Muller P, Igual J-P, Decoppet A, Eloit M, Pavio N (2007). Possible zoonotic transmission of hepatitis E from pet pig to its owner. Emerg Infect Dis.

[113] Colson P, Kaba M, Bernit E, Motte A, Tamalet C (2007). Hepatitis E associated with surgical training on pigs. Lancet.

[114] Pérez-Gracia MT, Mateos ML, Galiana C, Fernández-Barredo S, García A, Gómez MT, Moreira V (2007). Autochthonous hepatitis E infection in a slaughterhouse worker. Am J Trop Med Hyg.

[115] Gardinali NR, Barry AF, Otonel RAA, Alfieri AF, Alfieri AA (2012). Hepatitis E virus in liver and bile samples from slaughtered pigs of Brazil. Mem Inst Oswaldo Cruz.

[116] Traoré KA, Ouoba JB, Huot N, Rogée S, Dumarest M, Traoré AS, Pavio N, Barro N, Roques P (2015). Hepatitis E virus exposure is increased in pork butchers from Burkina Faso. Am J Trop Med Hyg.

[117] de Paula VS, Wiele M, Mbunkah AH, Daniel AM, Kingsley MT, Schmidt-Chanasit J (2013). Hepatitis E virus genotype 3 strains in domestic pigs, Cameroon. Emerg Infect Dis.

[118] Wilhelm B, Leblanc D, Houde A, Brassard J, Gagné M-J, Plante D, Bellon-Gagnon P, Jones TH, Muehlhauser V, Janecko N, Avery B, Rajić A, McEwen SA (2014). Survey of Canadian retail pork chops and pork livers for detection of hepatitis E virus, norovirus, and rotavirus using real time RT-PCR. Int J Food Microbiol.

[119] Li W, She R, Wei H, Zhao J, Wang Y, Sun Q, Zhang Y, Wang D, Li R (2009). Prevalence of hepatitis E virus in swine under different breeding environment and abattoir in Beijing, China. Vet Microbiol.

[120] Wenzel JJ, Preiss J, Schemmerer M, Huber B, Plentz A, Jilg W (2011). Detection of hepatitis E virus (HEV) from porcine livers in Southeastern Germany and high sequence homology to human HEV isolates. J Clin Virol.

[121] Chan MCW, Kwok K, Hung T-N, Chan PKS (2017). Molecular epidemiology and strain comparison between hepatitis E virus in human sera and pig livers during 2014–2016 in Hong Kong. J Clin Microbiol.

[122] Kulkarni MA, Arankalle VA (2008). The detection and characterization of hepatitis E virus in pig livers from retail markets of India. J Med Virol.

[123] Okano H, Takahashi M, Isono Y, Tanaka H, Nakano T, Oya Y, Sugimoto K, Ito K, Ohmori S, Maegawa T, Kobayashi M, Nagashima S, Nishizawa T, Okamoto H (2014). Characterization of sporadic acute hepatitis E and comparison of hepatitis E virus genomes in acute hepatitis patients and pig liver sold as food in Mie, Japan. Hepatol Res.

[124] Sasaki Y, Haruna M, Murakami M, Hayashida M, Ito K, Noda M, Yamada Y (2013). Prevalence of *Campylobacter* spp., *Salmonella* spp., *Listeria monocytogenes*, and hepatitis E virus in swine livers collected at an abattoir. Jpn J Infect Dis.

[125] Ishida S, Yoshizumi S, Ikeda T, Miyoshi M, Goto A, Matsubayashi K, Ikeda H (2012). Detection and molecular characterization of hepatitis E virus in clinical, environmental and putative animal sources. Arch Virol.

[126] Cantú-Martínez MA, Roig-Sagués AX, Cedillo-Rosales S, Zamora-Ávila DE, Avalos-Ramírez R (2013) Molecular detection of hepatitis E virus in pig livers destined for human consumption in the state of Nuevo Leon, Mexico. Salud Publica Mex 55:193–195. (in Spanish) 10.1590/s0036-36342013000200011 23546411

[127] Intharasongkroh D, Sa-Nguanmoo P, Tuanthap S, Thongmee T, Duang-In A, Klinfueng S, Chansaenroj J, Vongpunsawad S, Theamboonlers A, Payungporn S, Chirathaworn C, Poovorawan Y (2017). Hepatitis E virus in pork and variety meats sold in fresh markets. Food Environ Virol.

[128] Kaba M, Davoust B, Marié J-L, Colson P (2010). Detection of hepatitis E virus in wild boar (Sus scrofa) livers. Vet J.

[129] Motoya T, Nagata N, Komori H, Doi I, Kurosawa M, Keta T, Sasaki N, Ishii K (2016). The high prevalence of hepatitis E virus infection in wild boars in Ibaraki Prefecture, Japan. J Vet Med Sci.

[130] MEGA6: molecular evolutionary genetics analysis version 6.0. http://www.megasoftware.net/. Accessed 27 Apr 2017 10.1093/molbev/mst197 PMC3840312 24132122

