# Supplementary material for: Recent knowledge on hepatitis E virus in Suidae reservoirs and transmission routes to human
Source: Vet Res. 2017 Nov 21;48:78. doi: 10.1186/s13567-017-0483-9 (PMC5696788; doi:10.1186/s13567-017-0483-9)
Supplement: Supplementary file 1 — Additional file 1. Prevalence of HEV-specific antibodies and HEV RNA among wild boars. Data from the literature (as referenced) are summarized in the table according to the geographic location, year of sampling, type of specimen used and the genotype detected. [file 13567_2017_483_MOESM1_ESM.docx]

**Supplemental Table 1**. Prevalence of HEV-specific antibodies and HEV RNA among wild boars. Data from the literature (as referenced) are summarized in the table according to the geographic location, year of sampling, type of specimen used and the genotype detected.

| **Location** | | | **Year** | **HEV antibodies** | **HEV RNA** | **Specimen** | **Geno-**  **type** | **Refe-**  **rence** |
| --- | --- | --- | --- | --- | --- | --- | --- | --- |
| **AUSTRALIA** | | | | | | | | |
| **Australia** | North | | **-** | **17%** (15/59) | **-** | **-** | **-** | [39] |
| **ASIA** | | | | | | | | |
| **Japan** | Countrywide | | 2002-2004 | **8.6%** (3/35) | - | - | - | [40] |
|  | Countrywide | | 2004-2005 | **1.6%** (1/62) | **2.3%** (2/87) | Serum/liver | 3 | [69] |
|  | Ehime | Wild | 2001-2004 | **25.5%** (100/392) | **3.1%** (12/392) | Serum | 3 | [48] |
|  |  | Captive |  | **71.4%** (10/14) | **0%** (0/14) |  | - |  |
|  | Gunma | | 2004-2006 | **4.5%** (4/89) | **1.1%** (1/89) | Serum | 3 | [64] |
|  | Countrywide | | 2003-2010 | **8.1%** (41/507) | **3.3%** (19/578) | Serum/Liver | 3/4/6 | [4] |
|  | Yamaguchi | | 2010-2012 | **41.6%** (47/113) | **4.5%** (5/112) | Serum | 4 | [43] |
|  | Countrywide | | 2010-2013 | **11.4%** (58/511) | **4.2%** (24/566) | Serum/Liver | 3/4/6 | [42] |
|  | Ibraki | | 2013-2014 | **41.2%** (28/68) | **10.3%** (7/68) | Serum/Liver/Feces | 3 | [129] |
| **Thailand** | Central (captive) | | 2009 | - | **2%** (1/51) | Feces | 3 | [131] |
| **EUROPE** | | | | | | | | |
| **Belgium** | Wallonia | | 2010-2011 | **34%** (n=383) | **5.3%** (5/94) | Serum/Liver | 3 | [60] |
| **Estonia** | Countrywide | | 2013 | **17.2%** (81/471)^#^ | **16%** (13/81)^##^ | Serosanguinous meat juice | 3 | [54] |
| **France** | Southeast | | 2007-2008 | - | **2.5%** (7/285) | Liver | 3 | [128] |
|  | Countrywide | | 2000-2004 | **14%** (59/421) | - | - | - | [49] |
|  | Southwest | | 2010-2011 | - | **5.8%** (5/86) | Liver | - | [80] |
|  | Corsica | | 2009/2010/2012 | **26.1%** (74/284) | **1.8%** (5/284) | Liver/Bile | - | [55] |
| **Germany** | North | | 1995-1996 | - | **5.3%** (10/189) | Serum | 3 | [132] |
|  | Northeast/center | | 2005-2008 | - | **14.9%** (22/148) | Liver | 3 | [58] |
|  | Countrywide | | 2007 | **29.9%** (32/107) | **68.2%** (90/132) | Liver/Bile/Serum | 3 | [50] |
|  | Centre | | 2008 | - | **14.5%** (18/124) | Serum | 3 | [133] |
|  | North | | 1996-1997 | - | **3.4%** (32/955) | Blood | 3 | [134] |
|  |  |  | 2005-2006 | - | **5.2%** (3/58) | Blood | 3 |  |
|  |  |  | 2009-2010 | - | **10.4%** (14/134) | Liver | 3 |  |
|  | Centre | | 2012-2013 | **41.3%** (19/46) | **15.2%** (7/46) | Serum | 3 | [111] |
|  | - | | 2013-2015 | **45%** (81/180) | **16.8%** (39/232) | Serum/Liver | 3 | [56] |
| **Hungary** | Countrywide | | 2005-2009 |  | **10.7%** (8/75) | Liver | 3 | [78] |
| **Italy** | Northeast | | 2006 | - | **25%** (22/88) | Bile | 3 | [45] |
|  | Centre/North | | 2009-2012 | **10.2%** (226/2211) | **0%** (0/493)* | Liver | - | [51] |
|  | Northwest | | 2012-2013 | **4.9%** (29/594) | **3.7%** (12/320) | Liver | 3 | [52] |
|  | Centre/Northwest | | 2012-2014 | - | **1.9%** (7/372) | Liver | 3 | [62] |
|  | Centre | | - | **40.7%** (93/228) | **33.5%** (55/164) | Liver | 3 | [63] |
|  | Centre | | 2011-2012 | **56.2%** (36/64) | **9.4%** (6/64) | Feces | 3 | [61] |
|  | North/South | | 2012-2015 | - | **1.5%** (3/196) | Feces | 3 | [135] |
| **The Netherlands** | Centre | | 2005 | - | **3.8%** (1/26) | Feces | 3 | [136] |
|  | Centre/South | | 2005-2008 | **12%** (165/1029) | **7.5%** (8/106) | Serum/Feces/Liver/Muscle | 3 | [66] |
| **Poland** | Countrywide | | 2012-2013 | **44.4%** (116/261) | - | - | - | [53] |
|  | Northwest | | 2012-2014 | **17.2%** (28/163) | **28.8%** (47/163) | Serum/Feces | 3 | [137] |
| **Portugal** | Centre (captive) | | 2012 | - | **10%** (4/40) | Stool | 3 | [67] |
|  | Northeast/Centre | | 2011-2012 | - | **25%** (20/80) | Liver | 3 |  |
| **Slovenia** | Countrywide | | 2009-2011 | **30.2%** (87/288) | **0.35%** (1/288) | Serum | - | [138] |
| **Spain** | Centre/South | | 2000-2005 | **42.7%** (64/150) | **19.6%** (27/138) | Serum | 3 | [59] |
|  | Countrywide | | 2000-2011 | **26.3%** (n=942) | - | - | - | [57] |
|  | Centre | | 2003-2010 | **57.4%** (62/108) | **10.1%** (16/158) | Serum | 3 | [77] |
| **Sweden** | South | | - | - | **8.2%** (13/159) | Serum | 3 | [139] |
|  | South | | 2012-2015 | **8.2%** (11/134) | **7.9%** (11/139) | Serum/Feces | 3 | [68] |
| **Switzerland** | Countrywide | | 2008-2012 | **12.5%** (38/303) | - | - | - | [14] |

^#^ Seroprevalence determined using serosanguinous meat juice.

^##^ RNA prevalence determined using samples from anti-HEV-positive wild boars.

* 412/493 liver samples were analyzed clustered in 18 pools.

(-) Not determined or available.

4. Sato Y, Sato H, Naka K, Furuya S, Tsukiji H, Kitagawa K, Sonoda Y, Usui T, Sakamoto H, Yoshino S, Shimizu Y, Takahashi M, Nagashima S, Jirintai null, Nishizawa T, Okamoto H (2011) A nationwide survey of hepatitis E virus (HEV) infection in wild boars in Japan: identification of boar HEV strains of genotypes 3 and 4 and unrecognized genotypes. Arch Virol 156:1345–1358 . doi: 10.1007/s00705-011-0988-x

14. Burri C, Vial F, Ryser-Degiorgis M-P, Schwermer H, Darling K, Reist M, Wu N, Beerli O, Schöning J, Cavassini M, Waldvogel A (2014) Seroprevalence of hepatitis E virus in domestic pigs and wild boars in Switzerland. Zoonoses Public Health 61:537–544 . doi: 10.1111/zph.12103

39. Chandler JD, Riddell MA, Li F, Love RJ, Anderson DA (1999) Serological evidence for swine hepatitis E virus infection in Australian pig herds. Vet Microbiol 68:95–105

40. Sonoda H, Abe M, Sugimoto T, Sato Y, Bando M, Fukui E, Mizuo H, Takahashi M, Nishizawa T, Okamoto H (2004) Prevalence of Hepatitis E Virus (HEV) Infection in Wild Boars and Deer and Genetic Identification of a Genotype 3 HEV from a Boar in Japan. J Clin Microbiol 42:5371–5374 . doi: 10.1128/JCM.42.11.5371-5374.2004

42. Takahashi M, Nishizawa T, Nagashima S, Jirintai S, Kawakami M, Sonoda Y, Suzuki T, Yamamoto S, Shigemoto K, Ashida K, Sato Y, Okamoto H (2014) Molecular characterization of a novel hepatitis E virus (HEV) strain obtained from a wild boar in Japan that is highly divergent from the previously recognized HEV strains. Virus Res 180:59–69 . doi: 10.1016/j.virusres.2013.12.014

43. Hara Y, Terada Y, Yonemitsu K, Shimoda H, Noguchi K, Suzuki K, Maeda K (2014) High prevalence of hepatitis E virus in wild boar (Sus scrofa) in Yamaguchi Prefecture, Japan. J Wildl Dis 50:378–383 . doi: 10.7589/2013-06-144

45. Martelli F, Caprioli A, Zengarini M, Marata A, Fiegna C, Di Bartolo I, Ruggeri FM, Delogu M, Ostanello F (2008) Detection of hepatitis E virus (HEV) in a demographic managed wild boar (Sus scrofa scrofa) population in Italy. Vet Microbiol 126:74–81 . doi: 10.1016/j.vetmic.2007.07.004

48. Michitaka K, Takahashi K, Furukawa S, Inoue G, Hiasa Y, Horiike N, Onji M, Abe N, Mishiro S (2007) Prevalence of hepatitis E virus among wild boar in the Ehime area of western Japan. Hepatol Res Off J Jpn Soc Hepatol 37:214–220 . doi: 10.1111/j.1872-034X.2007.00030.x

49. Carpentier A, Chaussade H, Rigaud E, Rodriguez J, Berthault C, Boué F, Tognon M, Touzé A, Garcia-Bonnet N, Choutet P, Coursaget P (2012) High hepatitis E virus seroprevalence in forestry workers and in wild boars in France. J Clin Microbiol 50:2888–2893 . doi: 10.1128/JCM.00989-12

50. Adlhoch C, Wolf A, Meisel H, Kaiser M, Ellerbrok H, Pauli G (2009) High HEV presence in four different wild boar populations in East and West Germany. Vet Microbiol 139:270–278 . doi: 10.1016/j.vetmic.2009.06.032

51. Martinelli N, Pavoni E, Filogari D, Ferrari N, Chiari M, Canelli E, Lombardi G (2015) Hepatitis E virus in wild boar in the central northern part of Italy. Transbound Emerg Dis 62:217–222 . doi: 10.1111/tbed.12118

52. Caruso C, Modesto P, Bertolini S, Peletto S, Acutis PL, Dondo A, Robetto S, Mignone W, Orusa R, Ru G, Masoero L (2015) Serological and virological survey of hepatitis E virus in wild boar populations in northwestern Italy: detection of HEV subtypes 3e and 3f. Arch Virol 160:153–160 . doi: 10.1007/s00705-014-2246-5

53. Larska M, Krzysiak MK, Jabłoński A, Kęsik J, Bednarski M, Rola J (2015) Hepatitis E virus antibody prevalence in wildlife in Poland. Zoonoses Public Health 62:105–110 . doi: 10.1111/zph.12113

54. Ivanova A, Tefanova V, Reshetnjak I, Kuznetsova T, Geller J, Lundkvist Å, Janson M, Neare K, Velström K, Jokelainen P, Lassen B, Hütt P, Saar T, Viltrop A, Golovljova I (2015) Hepatitis E Virus in Domestic Pigs, Wild Boars, Pig Farm Workers, and Hunters in Estonia. Food Environ Virol 7:403–412 . doi: 10.1007/s12560-015-9210-8

55. Jori F, Laval M, Maestrini O, Casabianca F, Charrier F, Pavio N (2016) Assessment of Domestic Pigs, Wild Boars and Feral Hybrid Pigs as Reservoirs of Hepatitis E Virus in Corsica, France. Viruses 8: . doi: 10.3390/v8080236

56. Anheyer-Behmenburg HE, Szabo K, Schotte U, Binder A, Klein G, Johne R (2017) Hepatitis E Virus in Wild Boars and Spillover Infection in Red and Roe Deer, Germany, 2013-2015. Emerg Infect Dis 23:130–133 . doi: 10.3201/eid2301.161169

57. Boadella M, Ruiz-Fons JF, Vicente J, Martín M, Segalés J, Gortazar C (2012) Seroprevalence evolution of selected pathogens in Iberian wild boar. Transbound Emerg Dis 59:395–404 . doi: 10.1111/j.1865-1682.2011.01285.x

58. Schielke A, Sachs K, Lierz M, Appel B, Jansen A, Johne R (2009) Detection of hepatitis E virus in wild boars of rural and urban regions in Germany and whole genome characterization of an endemic strain. Virol J 6:58 . doi: 10.1186/1743-422X-6-58

59. de Deus N, Peralta B, Pina S, Allepuz A, Mateu E, Vidal D, Ruiz-Fons F, Martín M, Gortázar C, Segalés J (2008) Epidemiological study of hepatitis E virus infection in European wild boars (Sus scrofa) in Spain. Vet Microbiol 129:163–170 . doi: 10.1016/j.vetmic.2007.11.002

60. Thiry D, Mauroy A, Saegerman C, Licoppe A, Fett T, Thomas I, Brochier B, Thiry E, Linden A (2015) Belgian Wildlife as Potential Zoonotic Reservoir of Hepatitis E Virus. Transbound Emerg Dis. doi: 10.1111/tbed.12435

61. Mazzei M, Nardini R, Verin R, Forzan M, Poli A, Tolari F (2015) Serologic and molecular survey for hepatitis E virus in wild boar (Sus scrofa) in Central Italy. New Microbes New Infect 7:41–47 . doi: 10.1016/j.nmni.2015.05.008

62. Serracca L, Battistini R, Rossini I, Mignone W, Peletto S, Boin C, Pistone G, Ercolini R, Ercolini C (2015) Molecular Investigation on the Presence of Hepatitis E Virus (HEV) in Wild Game in North-Western Italy. Food Environ Virol 7:206–212 . doi: 10.1007/s12560-015-9201-9

63. Montagnaro S, De Martinis C, Sasso S, Ciarcia R, Damiano S, Auletta L, Iovane V, Zottola T, Pagnini U (2015) Viral and Antibody Prevalence of Hepatitis E in European Wild Boars (Sus scrofa) and Hunters at Zoonotic Risk in the Latium Region. J Comp Pathol 153:1–8 . doi: 10.1016/j.jcpa.2015.04.006

64. Sakano C, Morita Y, Shiono M, Yokota Y, Mokudai T, Sato-Motoi Y, Noda A, Nobusawa T, Sakaniwa H, Nagai A, Kabeya H, Maruyama S, Yamamoto S, Sato H, Kimura H (2009) Prevalence of hepatitis E virus (HEV) infection in wild boars (Sus scrofa leucomystax) and pigs in Gunma Prefecture, Japan. J Vet Med Sci 71:21–25

66. Rutjes SA, Lodder-Verschoor F, Lodder WJ, van der Giessen J, Reesink H, Bouwknegt M, de Roda Husman AM (2010) Seroprevalence and molecular detection of hepatitis E virus in wild boar and red deer in The Netherlands. J Virol Methods 168:197–206 . doi: 10.1016/j.jviromet.2010.05.014

67. Mesquita JR, Oliveira RMS, Coelho C, Vieira-Pinto M, Nascimento MSJ (2014) Hepatitis E Virus in Sylvatic and Captive Wild Boar from Portugal. Transbound Emerg Dis. doi: 10.1111/tbed.12297

68. Roth A, Lin J, Magnius L, Karlsson M, Belák S, Widén F, Norder H (2016) Markers for Ongoing or Previous Hepatitis E Virus Infection Are as Common in Wild Ungulates as in Humans in Sweden. Viruses 8: . doi: 10.3390/v8090259

69. Nishizawa T, Takahashi M, Endo K, Fujiwara S, Sakuma N, Kawazuma F, Sakamoto H, Sato Y, Bando M, Okamoto H (2005) Analysis of the full-length genome of hepatitis E virus isolates obtained from wild boars in Japan. J Gen Virol 86:3321–3326 . doi: 10.1099/vir.0.81394-0

77. Kukielka D, Rodriguez-Prieto V, Vicente J, Sánchez-Vizcaíno JM (2015) Constant Hepatitis E Virus (HEV) Circulation in Wild Boar and Red Deer in Spain: An Increasing Concern Source of HEV Zoonotic Transmission. Transbound Emerg Dis. doi: 10.1111/tbed.12311

78. Forgách P, Nowotny N, Erdélyi K, Boncz A, Zentai J, Szucs G, Reuter G, Bakonyi T (2010) Detection of hepatitis E virus in samples of animal origin collected in Hungary. Vet Microbiol 143:106–116 . doi: 10.1016/j.vetmic.2009.11.004

80. Lhomme S, Top S, Bertagnoli S, Dubois M, Guerin J-L, Izopet J (2015) Wildlife Reservoir for Hepatitis E Virus, Southwestern France. Emerg Infect Dis 21:1224–1226 . doi: 10.3201/eid2107.141909

111. Schielke A, Ibrahim V, Czogiel I, Faber M, Schrader C, Dremsek P, Ulrich RG, Johne R (2015) Hepatitis E virus antibody prevalence in hunters from a district in Central Germany, 2013: a cross-sectional study providing evidence for the benefit of protective gloves during disembowelling of wild boars. BMC Infect Dis 15:440 . doi: 10.1186/s12879-015-1199-y

128. Kaba M, Davoust B, Marié J-L, Colson P (2010) Detection of hepatitis E virus in wild boar (Sus scrofa) livers. Vet J Lond Engl 1997 186:259–261 . doi: 10.1016/j.tvjl.2009.08.008

129. Motoya T, Nagata N, Komori H, Doi I, Kurosawa M, Keta T, Sasaki N, Ishii K (2016) The high prevalence of hepatitis E virus infection in wild boars in Ibaraki Prefecture, Japan. J Vet Med Sci 77:1705–1709 . doi: 10.1292/jvms.15-0173

131. Wiratsudakul A, Sariya L, Prompiram P, Tantawet S, Suraruangchai D, Sedwisai P, Sangkachai N, Suksai P, Ratanakorn P (2012) Detection and phylogenetic characterization of hepatitis E virus genotype 3 in a captive wild boar in Thailand. J Zoo Wildl Med Off Publ Am Assoc Zoo Vet 43:640–644 . doi: 10.1638/2011-0217R1.1

132. Kaci S, Nöckler K, Johne R (2008) Detection of hepatitis E virus in archived German wild boar serum samples. Vet Microbiol 128:380–385 . doi: 10.1016/j.vetmic.2007.10.030

133. Oliveira-Filho EF, Bank-Wolf BR, Thiel H-J, König M (2014) Phylogenetic analysis of hepatitis E virus in domestic swine and wild boar in Germany. Vet Microbiol 174:233–238 . doi: 10.1016/j.vetmic.2014.09.011

134. Vina-Rodriguez A, Schlosser J, Becher D, Kaden V, Groschup MH, Eiden M (2015) Hepatitis E virus genotype 3 diversity: phylogenetic analysis and presence of subtype 3b in wild boar in Europe. Viruses 7:2704–2726 . doi: 10.3390/v7052704

135. Di Profio F, Melegari I, Sarchese V, Robetto S, Marruchella G, Bona MC, Orusa R, Martella V, Marsilio F, Di Martino B (2016) Detection and genetic characterization of hepatitis E virus (HEV) genotype 3 subtype c in wild boars in Italy. Arch Virol 161:2829–2834 . doi: 10.1007/s00705-016-2964-y

136. Rutjes SA, Lodder WJ, Lodder-Verschoor F, van den Berg HHJL, Vennema H, Duizer E, Koopmans M, de Roda Husman AM (2009) Sources of hepatitis E virus genotype 3 in The Netherlands. Emerg Infect Dis 15:381–387 . doi: 10.3201/eid1503.071472

137. Dorn-In S, Schwaiger K, Twarużek M, Grajewski J, Gottschalk C, Gareis M (2016) Hepatitis E Virus in Wild Boar in Northwest Poland: Sensitivity of Methods of Detection. Foodborne Pathog Dis. doi: 10.1089/fpd.2016.2194

138. Žele D, Barry AF, Hakze-van der Honing RW, Vengušt G, van der Poel WHM (2016) Prevalence of Anti-Hepatitis E Virus Antibodies and First Detection of Hepatitis E Virus in Wild Boar in Slovenia. Vector Borne Zoonotic Dis Larchmt N 16:71–74 . doi: 10.1089/vbz.2015.1819

139. Widén F, Sundqvist L, Matyi-Toth A, Metreveli G, Belák S, Hallgren G, Norder H (2011) Molecular epidemiology of hepatitis E virus in humans, pigs and wild boars in Sweden. Epidemiol Infect 139:361–371 . doi: 10.1017/S0950268810001342
