# Supplementary material for: Recent knowledge on hepatitis E virus in Suidae reservoirs and transmission routes to human
Source: Vet Res. 2017 Nov 21;48:78. doi: 10.1186/s13567-017-0483-9 (PMC5696788; doi:10.1186/s13567-017-0483-9)
Supplement: Supplementary file 2 — Additional file 2. Summary of reports on serological studies in people with occupational contact to animals. Data from published studies (as referenced) investigating the prevalence of anti-HEV antibodies in different types of exposed and control groups in different countries are summarized in the table. [file 13567_2017_483_MOESM2_ESM.docx]

**Supplemental Table 2**. Summary of reports on serological studies in people with occupational contact to animals. Data from published studies (as referenced) investigating the prevalence of anti-HEV antibodies in different types of exposed and control groups in different countries are summarized in the table.

| **Exposed group** | **prevalence** | **Control group** | **prevalence** | **Country** | **Reference** |
| --- | --- | --- | --- | --- | --- |
| Slaughterhouse workers, butchers, pig farmers swine veterinarians | 35/114 (30.7 %) | anonymous volunteers, matched by age, sex and region | 161/804 (19.9 %) | Portugal | [140] |
| Pig farm workers | 24/79 (30%) | Blood donors | 162/1200 (14%) | Norway | [141] |
| Swine farmers  Cattle farmers  Sheep farmers  Deer farmers  Fox farmers  Mink farmers  Poultry farmers | 57/116 (49.1%)  22/83 (26.5%)  20/65 (30.8%)  35/87 (40.2%)  21/69 (30.4%)  28/88 (31.8%)  26/92 (28.3%) | Control  Subjects, age- and gender-matched | 121/600 (20.2%) | China | [142] |
| Workers with occupational contact with swine | 4/69 (5.7%) | People without occupational contact with swine | 1/73 (1.3%) | Italy | [143] |
| Swine vet-  erinarians | 8/83 (9.6%) | Blood donors | 15/170 (8.8%) | Italy | [106] |
| Hunters | 26/126 (21%) | German Health Examination Survey for Adults | 731/4352 (16.8%) | Germany | [111] |
| Pork butchers | 76/100 (76%) | Blood donors | 43/90 (47.8%) | Burkina Faso | [116] |
| People with recent and past direct pig contact | 13/113 (11.5%) | People without recent and past direct pig contact | 5/126  (4.0%) | Germany | [144] |
| Pet Veterinarians | 7/373 (9.9%) | Matched general population | 16/120 (13.3%) | Portugal | [145] |
| Pig farm workers  Forestry workers | 134/306 (43.8%)  84/231 (36.4%) | Social and administrative staff, gardeners, traders, cereal growers | 84/322 (26.1%) | France | [109] |
| Pig farmers | 39/171 (22.8%) | individuals with no history of occupa-  tional contact with pigs on a farm | 79/342 (23.1%) | Thailand | [107] |
| Forest workers | 99/555 (17.8%) | Blood donors | 33/298 (11.1%) | Germany | [108] |
| living on subsistence family or commercial farms, working in slaughterhouses | 26/310 (8.4%) | Blood donors from urban area | 4/101 (4.0%) | Brazil | [146] |
| Slaughterers, meat inspectors, pig farmers, veterinarians | 30/106 (28.3%) | Age- and gender-matched blood donors | 18/116 (15.5%) | Germany | [147] |
| Persons in contact with pigs | 25/113 (11.8%) | Unexposed individuals | 4/100 (4%) | Spain | [148] |
| Swine farmers,  pig handlers, and swine veterinarians | 19/101 (18.8%) | Volunteers with no contact with swine | 4/97 (4.1%) | Spain | [149] |
| Wild boar hunters | 19/75 (25.3%) | Residents from the same location | 18/326 (5.5%) | Japan | [110] |

106. De Sabato L, Di Bartolo I, Montomoli E, Trombetta C, Ruggeri FM, Ostanello F (2016) Retrospective Study Evaluating Seroprevalence of Hepatitis E Virus in Blood Donors and in Swine Veterinarians in Italy (2004). Zoonoses Public Health. doi: 10.1111/zph.12332

107. Hinjoy S, Nelson KE, Gibbons RV, Jarman RG, Mongkolsirichaikul D, Smithsuwan P, Fernandez S, Labrique AB, Patchanee P (2013) A cross-sectional study of hepatitis E virus infection in healthy people directly exposed and unexposed to pigs in a rural community in northern Thailand. Zoonoses Public Health 60:555–562 . doi: 10.1111/zph.12030

108. Dremsek P, Wenzel JJ, Johne R, Ziller M, Hofmann J, Groschup MH, Werdermann S, Mohn U, Dorn S, Motz M, Mertens M, Jilg W, Ulrich RG (2012) Seroprevalence study in forestry workers from eastern Germany using novel genotype 3- and rat hepatitis E virus-specific immunoglobulin G ELISAs. Med Microbiol Immunol (Berl) 201:189–200 . doi: 10.1007/s00430-011-0221-2

109. Chaussade H, Rigaud E, Allix A, Carpentier A, Touzé A, Delzescaux D, Choutet P, Garcia-Bonnet N, Coursaget P (2013) Hepatitis E virus seroprevalence and risk factors for individuals in working contact with animals. J Clin Virol Off Publ Pan Am Soc Clin Virol 58:504–508 . doi: 10.1016/j.jcv.2013.08.030

110. Toyoda K, Furusyo N, Takeoka H, Murata M, Sawayama Y, Hayashi J (2008) Epidemiological study of hepatitis E virus infection in the general population of Okinawa, Kyushu, Japan. J Gastroenterol Hepatol 23:1885–1890 . doi: 10.1111/j.1440-1746.2008.05568.x

111. Schielke A, Ibrahim V, Czogiel I, Faber M, Schrader C, Dremsek P, Ulrich RG, Johne R (2015) Hepatitis E virus antibody prevalence in hunters from a district in Central Germany, 2013: a cross-sectional study providing evidence for the benefit of protective gloves during disembowelling of wild boars. BMC Infect Dis 15:440 . doi: 10.1186/s12879-015-1199-y

116. Traoré KA, Ouoba JB, Huot N, Rogée S, Dumarest M, Traoré AS, Pavio N, Barro N, Roques P (2015) Hepatitis E Virus Exposure is Increased in Pork Butchers from Burkina Faso. Am J Trop Med Hyg 93:1356–1359 . doi: 10.4269/ajtmh.15-0321

140. Teixeira J, Mesquita JR, Pereira SS, Oliveira RMS, Abreu-Silva J, Rodrigues A, Myrmel M, Stene-Johansen K, Øverbø J, Gonçalves G, Nascimento MSJ (2017) Prevalence of hepatitis E virus antibodies in workers occupationally exposed to swine in Portugal. Med Microbiol Immunol (Berl) 206:77–81 . doi: 10.1007/s00430-016-0484-8

141. Lange H, Øverbø J, Borgen K, Dudman S, Hoddevik G, Urdahl AM, Vold L, Sjurseth SK (2017) Hepatitis E in Norway: seroprevalence in humans and swine. Epidemiol Infect 145:181–186 . doi: 10.1017/S0950268816002144

142. Kang Y-H, Cong W, Zhang X-Y, Wang C-F, Shan X-F, Qian A-D (2017) Hepatitis E virus seroprevalence among farmers, veterinarians and control subjects in Jilin province, Shandong province and Inner Mongolia Autonomous Region, China. J Med Virol 89:872–877 . doi: 10.1002/jmv.24693

143. Caruso C, Peletto S, Rosamilia A, Modesto P, Chiavacci L, Sona B, Balsamelli F, Ghisetti V, Acutis PL, Pezzoni G, Brocchi E, Vitale N, Masoero L (2016) Hepatitis E Virus: A Cross-Sectional Serological and Virological Study in Pigs and Humans at Zoonotic Risk within a High-Density Pig Farming Area. Transbound Emerg Dis. doi: 10.1111/tbed.12533

144. Krumbholz A, Joel S, Dremsek P, Neubert A, Johne R, Dürrwald R, Walther M, Müller TH, Kühnel D, Lange J, Wutzler P, Sauerbrei A, Ulrich RG, Zell R (2014) Seroprevalence of hepatitis E virus (HEV) in humans living in high pig density areas of Germany. Med Microbiol Immunol (Berl) 203:273–282 . doi: 10.1007/s00430-014-0336-3

145. Mesquita JR, Valente-Gomes G, Conceição-Neto N, Nascimento MSJ (2014) Pet veterinarians have no increased risk of hepatitis E compared to the general population. J Med Virol 86:954–956 . doi: 10.1002/jmv.23927

146. Silva SMT da, Oliveira JM de, Vitral CL, Vieira K de A, Pinto MA, Souto FJD (2012) Prevalence of hepatitis E virus antibodies in individuals exposed to swine in Mato Grosso, Brazil. Mem Inst Oswaldo Cruz 107:338–341

147. Krumbholz A, Mohn U, Lange J, Motz M, Wenzel JJ, Jilg W, Walther M, Straube E, Wutzler P, Zell R (2012) Prevalence of hepatitis E virus-specific antibodies in humans with occupational exposure to pigs. Med Microbiol Immunol (Berl) 201:239–244 . doi: 10.1007/s00430-011-0210-5

148. Galiana C, Fernández-Barredo S, Pérez-Gracia MT (2010) [Prevalence of hepatitis E virus (HEV) and risk factors in pig workers and blood donors]. Enferm Infecc Microbiol Clin 28:602–607 . doi: 10.1016/j.eimc.2010.01.010

149. Galiana C, Fernández-Barredo S, García A, Gómez MT, Pérez-Gracia MT (2008) Occupational exposure to hepatitis E virus (HEV) in swine workers. Am J Trop Med Hyg 78:1012–1015
